# *Austromonticola*, a new genus of broad-nosed weevil (Coleoptera, Curculionidae, Entiminae) from montane areas of New Zealand

**DOI:** 10.3897/zookeys.707.12649

**Published:** 2017-10-10

**Authors:** Samuel D. J. Brown

**Affiliations:** 1 Bio-Protection Research Centre, PO Box 85084, Lincoln University 7647, Canterbury, New Zealand; 2 AgResearch, Gerald St, Lincoln, Canterbury, New Zealand

**Keywords:** Biodiversity, taxonomy, alpine, speciation, functional morphology

## Abstract

*Austromonticola* **gen. n.** is proposed for a group of eight New Zealand alpine broad-nosed weevil species, all of which are here described: *A. atriarius* **sp. n.** (type locality: Umbrella Mountains, Central Otago), *A. caelibatus* **sp. n.** (type locality: Ohau Range, Mackenzie), *A. furcatus* **sp. n.** (type locality: Old Man Range, Central Otago), *A. inflatus* **sp. n.** (type locality: Hawkdun Range, Central Otago), *A. planulatus* **sp. n.** (type locality: St Marys Range, Central Otago), *A. postinventus* **sp. n.** (type locality: Kirkliston Range, South Canterbury), *A. mataura* **sp. n.** (type locality: Mt Dick, Otago Lakes) and *A. rotundus* **sp. n.** (type locality: Old Man Range, Central Otago). All species occur exclusively above 1000 m elevation in the mountains of Central Otago and South Canterbury in the South Island. A phylogeny of the genus, including six outgroups, was inferred from 33 morphological characters. It resolved the genus as monophyletic, and revealed two strongly supported clades within *Austromonticola*. DNA sequences of four gene regions were obtained from five species. Of these, the 3' end of COI proved to be the most suitable for the identification of specimens. Females of all species have diagnostic secondary sexual structures on the elytra and ventrites. These structures are hypothesised to have evolved to assist with oviposition in and beside cushion plants or by selection for structures to mitigate the costs to females of prolonged mating.

## Introduction

The indigenous entimine weevil fauna of New Zealand currently consists of 28 described genera, containing 247 species. Taxonomic research on these weevils, especially at the genus level, has been dominated by the works of Francis Polkinghorne Pascoe (1875, 1877, 1876a, 1876b), Thomas Broun (1880, 1881, 1886, 1903, 1909a, 1909b, 1911, 1913, 1915, 1921) and David Sharp (1886) in the late 19th and early 20th centuries. Since then, few additional species have been described (Marshall 1926, 1931, 1937; Barratt and Kuschel 1996), and—with the exception of several generic synonyms proposed by Kuschel (1964, 1969, 1972, 1982)—the composition of most New Zealand entimine weevil genera has remained largely unmodified since Broun's (1921) last work on the group. Recent research, however, indicates that understanding of the genus diversity of broad-nosed weevils in New Zealand has been obscured by imprecise and polyphyletic generic concepts (Brown 2017), and many species and genera remain undescribed. This paper describes a new genus of entimine weevils that is restricted to high-alpine vegetation types and whose females exhibit exaggerated ornamentation on the abdominal ventrites.

The mountains of New Zealand are some of the most dramatic and recognisable landscapes of the country. Areas above 1000 m in elevation form a significant proportion of the available land area in the South Island. Geological evidence reveals that these landscapes have been formed relatively recently, with most ranges only appearing in the past five million years (Youngson et al. 1998; Craw et al. 2012). Despite this youth, these alpine regions harbour a rich flora and fauna, which are both endemic to New Zealand and restricted to alpine areas (Mark 2012). The alpine endemic biota include plants (McGlone et al. 2001), birds (Michelsen-Heath and Gaze 2007), lizards (Whitaker 1984; Bell and Patterson 2008), beetles (Leschen and Buckley 2015; Seago et al. 2015), moths (Gaskin 1975; Hoare 2012), cicadas (Buckley and Simon 2007; Dugdale and Fleming 1978), cockroaches (Chinn and Gemmell 2004) and Orthoptera (Trewick et al. 2000; Trewick 2008). Resolving this paradox of distinctive and highly endemic biota in a recent landscape has been a research priority in recent decades (Heenan and McGlone 2013; Buckley and Simon 2007; Winkworth et al. 2005).

## Materials and methods

Field collected specimens were killed in 100% ethanol or placed directly into a freezer at -20°C. Ethanol-preserved specimens were used preferentially for DNA extraction and sequencing.

Genitalia were examined by softening specimens for a short time in warm water, before removing the abdomen by inserting fine forceps between the metaventrite and ventrite 1. The abdomen was digested in porcine pancreatin enzyme solution for c. 36 h (Álvarez Padilla and Hormiga 2008), the lysate of which was subsequently used for DNA extraction. If specimens had not cleared satisfactorily at the end of this time, or were unsuitable for DNA extraction, abdomens were digested in room-temperature 10g/l KOH for up to two hours.

After clearing, the abdomen was flayed by cutting down the right side of the abdomen with spring scissors. Male genitalia were removed by severing the strong ligaments connecting sternite 8 to tergite 8, then cutting through the pretegminal membrane between the phallobase and the anus. Female genitalia were stained briefly by immersion in a 1g/l solution of Chlorazol Black in 70% ethanol, then removed by cutting through the membranes connecting tergites 7 and 8. Sternite 8 and tergite 8 were separated from the gonocoxites by cutting through their connecting membranes. Genitalia were photographed, then mounted on a card using dimethyl hydantoin formaldehyde (DMHF) (Liberti 2005), which was then pinned below the specimen.

Genitalia illustrations were prepared from photographs, using the program Inkscape (v. 0.91, Inkscape Team 2004-2017). Other line drawings were made with a Zeiss Stemi SV6 stereo microscope fitted with a camera lucida. These drawings were scanned and inked digitally in Inkscape. Habitus photographs were taken using a Nikon DS-Ri1 microscope fitted with a digital camera and a mechanical z-stepper. The program Nikon NIS Elements v. 4.10 was used to prepare the image stack and to produce the final montaged image.

Terminology follows Oberprieler et al. (2014), Lawrence et al. (2010) and Wanat (2007). Body length was measured in lateral view, from the anterior margin of the eyes to the apex of the elytra. Rostrum width was measured across the antennal insertions in dorsal view. Legs are described in their idealised laterally extended position, thereby having dorsal, ventral, anterior and posterior surfaces. Everted ovipositors were measured from the centre of the ovipositor level with the apices of sternite 8 and tergite 8, to the apex of the gonocoxites. Pappolepidia (Brown 2017) are multiply finely divided scales (Fig. 114, “multifid hairs” of Kuschel 1969), found in abundance on the abdominal and thoracic ventrites of some species. The term ‘dolabriform’ is used to describe relatively short, broad scales that have a similar shape to an adze blade (Torre-Bueno 1979).

Descriptions of colour follow the terminology provided by the National Bureau of Standards (Kelly and Judd 1976). The NBS centroid colours are a comprehensive dictionary of colours, with natural-language descriptions. Digital representations of these colours have been provided by Jaffer (2011). The difference in colour contrast between elongate setiform scales ('setae') and their surrounding appressed scales is given using the rough descriptors `pale', `concolorous' and `dark'.

Specimens were prepared for scanning electron microscopy (SEM) by separating the abdomen from the specimen, removing the tergites and genitalia and brushing down the sternites. Specimens were then air-dried before being mounted with double-sided carbon tape onto aluminium SEM stubs (11 mm high, 12 mm diameter). Specimens were coated with gold using a Emitech K975X sputter coater. Photographs were taken using a JEOL JSM-7000F field emission scanning electron microscope (JEOL, Tokyo, Japan), with an accelerating voltage of 3 kV.

Specimens were obtained and deposited in the following collections:

**AMNZ** Auckland War Memorial Museum, Auckland, New Zealand

**ANIC** Australian National Insect Collection, CSIRO, Canberra, Australia

**CMNZ** Canterbury Museum, Christchurch, New Zealand

**IACC** Invermay Agricultural Centre Collection, Mosgiel, New Zealand

**LUNZ** Lincoln University Entomology Research Museum, Lincoln, Canterbury, New Zealand

**MONZ** Te Papa Tongarewa, National Museum of New Zealand, Wellington, New Zealand

**NHM** The Natural History Museum, London, United Kingdom

**NZAC** New Zealand Arthropod Collection, Manaaki Whenua Landcare Research, Tamaki, Auckland, New Zealand

**USNM** Smithsonian Institution National Museum of Natural History, Washington D.C., United States of America

Label data of holotypes are transcribed using the following conventions. Data of individual labels are enclosed using quotes (‘…’), lines are indicated with a solidus (/) and metadata are given in square brackets ([…]).

Two-letter area codes follow the bioregionalisation system proposed by Crosby et al. (1998). The following codes are used in this paper; CO (Central Otago), MK (Mackenzie), OL (Otago Lakes), SC (South Canterbury). Coordinates given after the locality names are in the WGS84 datum. Coordinates tagged “R” (Recorded) were obtained from coordinates on the label when given or from consultation with the collector. Coordinates tagged “A” (Approximate) were determined by using available gazetteers, primarily the New Zealand Gazetteer of Place Names (Land Information New Zealand 2016). These georeferenced data were used to extract estimated elevations from 25 m resolution digital elevation models of New Zealand provided by Landcare Research (Landcare Research 2010a, 2010b).

A generative conception of species (Wilkins 2009) is followed, where morphological data are used as the primary criteria to justify inclusion within each species. Species are defined by character sets that allow differentiation between groups that form diagnosable entities. These taxa are recognised as phenomena that require explanation through further evolutionary and ecological study (Wilkins 2010).

### Data resources

Occurrence data from the specimens examined in this paper are deposited at GBIF, the Global Biodiversity Information Facility, http://ipt.pensoft.net/resource.do?r=austromonticoladistribution. DNA alignments and analysis scripts are available from FigShare, http://dx.doi.org/10.6084/m9.figshare.5367457

### DNA sequencing and analysis

Only freshly collected specimens were used for sequencing. Genomic DNA was extracted from the pancreatin lysate (see above) using the Zymo Quick g-DNA Miniprep Kit (Zymo Research Corporation, Irvine, CA, U. S. A.), following the manufacturer’s instructions for a proteinase k extraction. Four gene regions were sequenced: the cytochrome *c* oxidase subunit I (COI) mitochondrial gene, the D2-D3 region of the 28S ribosomal RNA gene, the nuclear protein-coding gene arginine kinase (ArgK) and the nuclear protein-coding carbamoyl-phosphate synthetase 2-aspartate transcarbamylase-dihydroorotase (CAD) gene.

DNA was amplified using a 25 μl polymerase chain reaction (PCR) consisting of 1.25 U iStar Taq (iNtRON Biotechnology, Seongnam, South Korea), 0.4 mM dNTP, 1.5 mM MgCl2 and 0.2 μM of forward and reverse primers (Table 1). The COI primer combination LCO1490-JJ/TL2-N-3014 was used preferentially in order to amplify the whole gene, which was then sequenced using all four primers. If amplification using this combination was unsuccessful, C1-J-2183/TL2-N-3014 was used. Reactions were run on a C1000 Touch thermal cycler (Bio-Rad Laboratories Inc., Hercules, CA, USA) or a MJ Mini thermal cycler (Bio-Rad Laboratories Inc.) with an initial denature at 94 °C for 2 min, followed by 40 cycles at 94 °C (20 s), variable annealing temperature (20 s) and 72 °C (60 s), and with a final extension at 72 °C for 5 min. Annealing temperatures were 45 °C for COI, and 52 °C for 28S reactions. ArgK and CAD reactions were amplified using a touchdown protocol, with annealing temperatures starting at 50 °C, decreasing by 1 °C per cycle for 5 cycles, followed by 35 cycles at 45 °C. Purified PCR products were sequenced by Macrogen (Seoul, Korea) using ABI BigDye 3.1 technology on an ABI3730XL platform (Applied Biosystems).

**Table 1.** Markers and PCR primer combinations used in this research.

**Marker**	**Primer name**	**Direction**	**Primer sequence**	**Reference**
COI	C1-J-2183	Forward	5'-CAA CAT TTA TTT TGA TTT TTT GG-3'	Simon et al. 1994
	LCO1490-JJ	Forward	5'-CHA CWA AYC ATA AAG ATA TYG G-3'	Astrin and Stüben 2008
	HCO2198-JJ	Reverse	5'-AWA CTT CVG GRT GVC CAA ARA ATC A-3'	Astrin and Stüben 2008
	TL2-N-3014	Reverse	5'-TCC AAT GCA CTA ATC TGC CAT ATT A-3'	Simon et al. 1994
28S	S3660	Forward	5'-GAG AGT TMA ASA GTA CGT GAA AC-3'	Sequeira et al. 2000
	28S-Ff	Reverse	5'-TTA CAC ACT CCT TAG CGG AT-3'	Gómez-Zurita et al. 2005
ArgK	ArgKforB4	Forward	5'-GAY CCC ATC ATC GAR GAC TAC C-3'	McKenna et al. 2009
	ArgKrevB1	Reverse	5'-TCN GTR AGR CCC ATW CGT CTC-3'	McKenna et al. 2009
CAD	CADfor4	Forward	5'-TGG AAR GAR GTB GAR TAC GAR GTG GTY CG-3'	Jordal et al. 2011
	CADrev1mod	Reverse	5'-GCC ATY RCY TCB CCY ACR CTY TTC AT-3'	Jordal et al. 2011

Sequences were aligned by eye in Seaview (version 4.5.4) (Guoy et al. 2010). Uncorrected genetic distances (*p*-distances) were calculated using Ape (version 3.5) (Paradis et al. 2004), which were then decomposed into interspecific and intraspecific components using Spider (version 1.4-2) (Brown et al. 2012). Diagnostic nucleotides (Sarkar et al. 2008) for each species were identified using Spider.

### Morphological phylogenetic analysis

A total of 33 morphological characters were scored for 14 species (Table 2), including six outgroup taxa: *Irenimus parilis* Pascoe, 1876, *Brachyolus punctatus* White, 1846, *Inophloeus sulcifer* Broun, 1886, *Zenagraphus metallescens* Broun, 1915, “*Inophloeus*” *sternalis* Broun, 1904, and an undescribed genus and undescribed species represented by specimens collected from Chancellor Hut, Fox Glacier, Westland Te Poutini National Park. Specimens of this last taxon have been deposited in NZAC with specimen numbers IRE7143, IRE7144, IRE7145 and IRE7147.

The phylogenetic matrix was prepared using Mesquite (version 3.10) (Maddison and Maddison 2016). Parsimonious cladograms were inferred using the parsimony ratchet (Nixon 1999), as implemented in Phangorn (version 2.0.4) (Schliep 2011), using Fitch parsimony with a random starting tree and subtree pruning and regrafting (SPR) rearrangements. The ratchet was run 100 times to ensure thorough sampling of the treespace. Bootstrap and jackknife (delete-half method, Felsenstein 2004) support values were calculated using Phangorn with 100 replicates each. Due to *A. planulatus*, *A. caelibatus* and *A. postinventus* not having suitable specimens available for DNA sequencing, morphological and sequence data were not combined.

## Taxonomic treatment

### Austromonticola

Taxon classificationAnimaliaColeopteraCurculionidae

Brown gen. n.

http://zoobank.org/51010275-E6EE-47B9-B84B-1D868054AD07

#### Type species.

*Austromonticola mataura* new species, here designated. Gender: masculine.

#### Diagnosis.

Integument densely covered with small, grey appressed scales, elongate setiform scales ('setae') conspicuous along elytral interstriae. Rostrum stout, in dorsal view about 1.5 times longer than wide; subparallel proximally; scrobes lateral; ventral curvature with head capsule approximately 90°. Pronotum in dorsal view evenly convex. Elytra with small, shallow punctures, interstriae flat. Metanepisternal sutures complete. Metatibiae with apex simple. Penis tubular. Bursa copulatrix with a single sclerite.

#### Differential diagnosis.

The combination of characters given above allows separation of *Austromonticola* from all other New Zealand weevils. The complete metanepisternal sutures distinguish them from *Chalepistes* Brown, 2017, in which the sutures are lacking. The abrupt 90° deflexion of the rostrum distinguish them from *Catoptes* Schönherr, 1842, which has a smoothly deflexed rostrum, angled about 120° with the ventral surface of the head capsule. The ridged appressed scales, conspicuous setae, evenly convex pronotum and small strial punctures separate *Austromonticola* from species of *Inophloeus* Pascoe, 1875 and *Zenagraphus* Broun, 1915, which have smooth appressed scales, inconspicuous setae, sculptured pronota and large, deep strial punctures. The subparallel rostrum and lateral scrobes, distinguish *Austromonticola* from *Nicaeana* Pascoe, 1877 and *Haplolobus* Broun, 1893, which have proximally widening rostra and dorsally situated scrobes.

#### Description.

Body length ranging from 3.4 mm to 8.9 mm. Densely covered with appressed scales on all surfaces, interspersed with elongate setiform scales ('setae'); appressed scales on dorsum oval, 35–55 μm long, ridges visible at 30 × magnification, generally coloured bluish grey, brownish grey or blackish grey, easily abraded. **Rostrum**. Subparallel proximally in dorsal view, widened at antennal insertions. Epistome punctate, plurisetose, slightly raised above frons but separation indistinct. Epifrons with longitudinal median carina, lacking sulci; continuous with occiput, without distinct dorsal separation between head capsule and rostrum. **Antennae**. Sockets dorsolateral, situated in apical 1/3 of rostrum. Scapes clavate, reaching posterior margin of eye in repose. Funicular segments clavate, subspherical or oblately spheroid, moderately to loosely articulated, segments 7 almost as wide as club. Clubs two times longer than wide, tapering apicad. **Head capsule**. Interocular width in dorsal view greater than width of rostrum at base. Eyes large, lateral, flat, ovate to subcircular with long axis vertical, parallel with sagittal axis. Ventral curvature of head capsule and rostrum in lateral view angulate, approximately 90°. **Pronotum**. Disc in dorsal view smooth, evenly convex. Postocular lobes poorly to well developed; fringed with numerous short vibrissae attaining a maximum length of 1/3 times anterior-posterior length of eye. **Elytra**. In dorsal view approximately parallel-sided in anterior 2/3. Setae arising from interstriae. Elytral declivity in lateral view rounded in males, but sutural margin at top of declivity developed into tubercles in females of several species. Interstriae 3 above the declivity slightly swollen in both sexes of most species, interstriae 5 above the declivity rarely swollen. Ventral margin in lateral view sinuous, highest point near level of metacoxae. **Thorax**. Procoxae contiguous. Prosternum visible behind procoxae as a raised tubercle (“prosternellum”). Metaventrite with median suture visible only as a small, circular fovea posteriorly. Metanepisternal sutures complete. **Abdomen**. Ventrites 1 and 2 fused, subequal in length in middle; ventrites 3 and 4 subequal in length, approximately 0.5 times shorter than 1 or 2; ventrite 5 approximately equal in length to 1 or 2. Suture separating ventrites 1 and 2 curved anteriad in middle, other sutures straight. **Wings**. Absent. **Legs**. Uniformly and densely covered with appressed scales and setae, except for the posterior surface of the metafemora. Femora unarmed, maximum girth at about distal quarter. Pro- and mesotibiae with indistinct denticles along ventral margin and mucrones at apex; protibiae wider in distal 1/3 than proximal 1/3, incurved at apex. Metatibiae with dorsal and ventral margins subparallel; apical setal comb arcuate, pale; mucrones small, inconspicuous; without corbel. Tarsi with long, coarse setae on dorsal surface, without appressed scales; underside of segments 1 to 3 with short, dense setae forming pads medially divided by an inconspicuous glabrous line. Claws simple, separate, diverging. **Male genitalia**. Hemisternites 8 fully separate, with a forked membranous sclerite on the anterior margin of the membrane connecting them (‘spiculum relictum’, Thompson 1992; Wanat 2007; Franz and Cardona-Duque 2013). Penis with pedon tubular, strongly curved, lateral lobes meeting or narrowly separated dorsally; temones shorter than pedon. Endophallus moderate in length, usually reaching anterior 1/3 of temones when in repose; armed with a variably-shaped sclerite surrounding the primary gonopore ('gonoporial sclerite'), other sclerites variably present. Tegmen with ring complete; parameroid lobes moderately developed, 0.35 times length of manubrium (Figs 85, 86); manubrium shorter than temones. **Female genitalia**. Sternite 8 with spiculum ventrale more than twice as long as blade. Gonocoxites divided into two parts; proximal part about 2.3 times longer than distal part, largely unsclerotised except for a strongly sclerotised rod; rods ventrally situated, broadening at proximal end; distal gonocoxite moderately sclerotised. Bursa copulatrix with a single sclerite.

#### Distribution.

Restricted to alpine regions in Otago and South Canterbury, New Zealand.

#### Etymology.

Derived from the Latin *australis*, meaning ‘southern’ and *monticola*, meaning ‘mountain dweller’, alluding to the habitat of the species of this genus, being confined to the mountains of the southern part of the South Island. Gender masculine.

#### Biology.

Specimens of the genus have been collected in fellfield and cushionfield vegetation communities (Mark, 2012), commonly on top of, and close beside, cushion plants of the genera *Phyllachne* J. R. et G. Forst., 1776 (Stylidiaceae), *Scleranthus* L., 1753 (Caryophyllaceae), *Veronica* L., 1753 (Plantaginaceae), *Hectorella* Hook. f., 1864 (Montiaceae), *Dracophyllum* Labill., 1798 (Ericaceae) and *Raoulia* Hook. f., 1846 (Asteraceae), particularly when the plants have been in flower. Some species have also been found under specimens of *Celmisia* Cass., 1825 (Asteraceae) and *Geum* L., 1753 (Rosaceae). The larvae are as yet unknown.

Most specimens have been collected by hand collecting, though some have been captured in pitfall traps or by heat extraction from litter and turf samples.

### Austromonticola atriarius

Taxon classificationAnimaliaColeopteraCurculionidae

Brown sp. n.

http://zoobank.org/3E3220A4-9418-4B2E-A080-F7E48B50FBF2

Figs 1 , 2 , 17 , 18 , 39 , 40 , 41 , 42 , 43 , 44 , 45 , 100 , 101 , 116

#### Diagnosis.

Body size medium, 4 mm in length. Pronotum with median furrow. Elytral declivity in females with sutural tubercle; margin of ventrite 4 produced into a lamina, with bifurcate median process (Fig. 100); margin of ventrite 5 with a slim horn on either side of genital opening (Fig. 100).

#### Description.

Body length 3.60 mm to 4.55 mm (*X*‒ = 4.19 mm, *s* = 0.36, *n* = 8). Integument black. Dorsum densely covered with moderate yellowish brown to greyish brown appressed scales, pale “V” on elytral declivity often present; pronotum same colour as elytra, with pale posterolateral maculae lining up with pale maculae on humeral angles of elytra, especially prominent in females. Femora and tibiae with dense appressed scales concolorous with elytral scales, usually with pale band in distal 1/4 of femora. Tarsi with integument deep orange. **Rostrum**. Length 0.72 mm to 0.96 mm (*X*‒ = 0.85 mm, *s* = 0.09, *n* = 7), width 0.52 mm to 0.65 mm (*X*‒ = 0.59 mm, *s* = 0.05, *n* = 7), length/width ratio 1.33 to 1.55 (*X*‒ = 1.44, *s* = 0.07, *n* = 7). Epifrons with appressed scales imbricate; setae dolabriform, decumbent, dark; median and lateral carinae not evident. Dorsal carinae arched over antennal insertions. Lateral area ventral of antennal insertions with thick setae, without appressed scales. **Antennae**. Scapes in repose reaching hind margin of eye; covered with appressed scales and setae. Funicular segments moderately articulated; segments 1 clavate, subequal in length to 2; segments 2 clavate, about two times longer than 3; segments 3 to 4 clavate; segments 5 to 7 oblately spheroid. **Pronotum**. Length 1.07 mm to 1.29 mm (*X*‒ = 1.16 mm, *s* = 0.09, *n* = 7), width 1.58 mm to 2.08 mm (*X*‒ = 1.88 mm, *s* = 0.20, *n* = 7), length/width ratio 0.77 to 0.97 (*X*‒ = 0.87, *s* = 0.07, *n* = 7); in dorsal view widest in anterior 1/4, lateral margins evenly curved. Anterior margin slightly emarginate medially, posterior margin straight. Disc in dorsal view evenly convex, with median furrow; appressed scales imbricate; setae dolabriform to claviform, decumbent, dark to concolorous. Postocular lobes moderately developed. **Elytra**. Length 2.40 mm to 3.09 mm (*X*‒ = 2.78 mm, *s* = 0.26, *n* = 7), width 1.58 mm to 2.08 mm (*X*‒ = 1.88 mm, *s* = 0.20, *n* = 7), length/width ratio 1.39 to 1.61 (*X*‒ = 1.48, *s* = 0.08, *n* = 7). Anterior margin curved posteriad in middle, humeral angles rounded. Appressed scales imbricate. Setae claviform, decumbent, concolorous. Striae moderately impressed; interstriae slightly convex. Interstriae 1 at declivity flat in males, produced into a tubercle in females. Interstriae 3 and 5 at declivity swollen in both sexes. Apex in lateral view square in males; produced ventrad in females. **Thoracic ventrites**. Mesoventral process rounded. Mesanepisterna, mespimera, metanepisterna and metaventrite densely clothed with pappolepidia. **Abdomen**. Ventrites sparsely clothed, appressed scales most numerous medially, pappolepidia becoming dominant laterally. Apex rounded. Males with ventrite 1 strongly depressed medially; ventrite 5 flat. Females with ventrite 1 flat; ventrite 4 with posterior margin produced medially into a bifurcated lamina (Figs 100, 101); ventrite 5 with median furrow, posterior margin broadly emarginate with a strong horn on either side of emargination. **Male genitalia**. Figs 39, 40. Penis with apex acute, upturned; ostial region thickened, forming a crest. Endophallus with lightly sclerotised plate proximally from primary gonopore, gonoporial sclerite large, with distinct posterior lobes. Temones 0.72 times as long as pedon. **Female genitalia**. Figs 41–45. Distal gonocoxites slender, 2.7 times longer than high. Bursa copulatrix long; not constricted anteriorly of proximal gonocoxite; sclerite horseshoe-shaped, squat. Sternite 8 narrowly rounded apically, membranous laterally. Everted ovipositor 3.44 mm in length, 0.75 times body length.

#### DNA sequences. COI.

KX191432. **28S**. KX192009. **ArgK**. KX191719. **CAD**. KX191161.

#### Type material examined.

**Holotype**. Female (NZAC). Specimen mounted on card teardrop; abdomen removed, dissected and mounted in DMHF on white card below specimen; otherwise entire; elytra parted at apex. Labelled ‘NEW ZEALAND CO / Gem Lake / Umbrella Mountains / 10 Feb 2014 / SDJ Brown’ [printed, cream card], ‘On *Phyllachne* cushion / 1430 m / 45.5703°S, 169.1021°E [printed, cream card], ‘*Irenimus* taxonomy / and systematics / SDJ Brown / PhD Thesis 2012–2015 / IRE4875’ [printed, cream card], ‘HOLOTYPE / *Austromonticola* / *atriarius* / Brown 2017’ [printed, red card]. Genomic DNA extract from enzyme digestion of abdomen: E300 (NZAC). CAD sequence KX191167; COI sequence KX191438; ArgK sequence KX191722; 28S sequence KX192015.

**Paratypes**. A total of 7 specimens (4 males, 3 females) designated as paratypes, bearing blue paratype label. Paratype specimens deposited in NHM, IACC, LUNZ, NZAC.

**CO.** Gem Lake [45°34.236'S, 169°6.384'E, A], 14–15 Dec 1985, Barratt BIP, 1300 m (NHM: 1); Gem Lake [45°34.236'S, 169°6.384'E, A], 14–15 Dec 1985, Barratt BIP, 1400 m (IACC: 1); Gem Lake [45°34.236'S, 169°6.384'E, A], 15 Dec–15 Jan 1986, Barratt BIP, 1430 m (NHM: 1, IACC: 1, LUNZ: 2, NZAC: 1).

#### Distribution.

Fig. 116. South Island: **CO**: Umbrella Mountains.

#### Elevational range.

Label data: 1300 m to 1430 m (*X*‒ = 1410 m, *s* = 46, *n* = 8). Georeferenced data: 1297 m to 1423 m (*X*‒ = 1313 m, *s* = 44, *n* = 8).

#### Etymology.

From the Latin noun *atriarius*, ‘porter, doorkeeper’, in reference to the armature surrounding the female genital opening and alluding to a possible function of preventing unwanted mating attempts. The name is a noun in apposition.

#### Biology.

Found in cushionfield, with a single specimen recorded in association with *Phyllachne*.

### Austromonticola caelibatus

Taxon classificationAnimaliaColeopteraCurculionidae

Brown sp. n.

http://zoobank.org/423B4D86-6AD7-4214-8A57-0B21CC7670B0

Figs 15 , 16 , 46 , 47 , 48 , 49 , 115

#### Diagnosis.

Body size large, 8 mm in length. Epifrons flat, with semi-erect setae. Funicle segments 7 subconical. Pronotum evenly convex. Elytra with erect, piliform setae.

#### Description.

Body length 7.83 mm to 8.91 mm (*X*‒ = 8.28 mm, *s* = 0.41, *n* = 5). Integument black. Dorsum densely covered with fine blackish blue appressed scales without metallic reflections. Femora and tibiae with appressed scales, unicolorous, concolorous with elytral scales. Tarsi with integument blackish red. **Rostrum**. Length 1.60 mm to 1.72 mm (*X*‒ = 1.65 mm, *s* = 0.05, *n* = 5), width 0.80 mm to 1.00 mm (*X*‒ = 0.94 mm, *s* = 0.08, *n* = 5), length/width ratio 1.68 to 2.04 (*X*‒ = 1.77, *s* = 0.15, *n* = 5). Epifrons with appressed scales tessellate; setae piliform, semi-erect, concolorous; median and lateral carinae evident. Dorsal carinae arched over antennal insertions. Lateral area ventral of antennal insertions with fine setae and appressed scales. **Antennae**. Scapes in repose reaching beyond hind margin of eyes; covered with appressed scales and setae. Funicular segments loosely articulated; segments 1 clavate, roughly as long as 2; segments 2 clavate, about two times longer than 3; segments 3 to 6 clavate, getting progressively shorter; segments 7 subconical. **Pronotum**. Length 1.90 mm to 2.24 mm (*X*‒ = 2.05 mm, *s* = 0.13, *n* = 5), width 3.08 mm to 3.25 mm (*X*‒ = 3.16 mm, *s* = 0.07, *n* = 5), length/width ratio 0.88 to 1.02 (*X*‒ = 0.94, *s* = 0.05, *n* = 5); in dorsal view widest in anterior 1/3, lateral margins evenly curved. Anterior margin entire, posterior margin straight. Disc in dorsal view evenly curved; appressed scales tessellate; setae piliform, semi-erect, concolorous. Postocular lobes moderately developed. **Elytra**. Length 5.15 mm to 5.61 mm (*X*‒ = 5.36 mm, *s* = 0.23, *n* = 5), width 3.08 mm to 3.25 mm (*X*‒ = 3.16 mm, *s* = 0.07, *n* = 5), length/width ratio 1.63 to 1.76 (*X*‒ = 1.70, *s* = 0.05, *n* = 5). Anterior margin slightly curved posteriad in middle, humeral angles rounded. Appressed scales tessellate. Setae piliform, semi-erect to erect, concolorous on disc, pale laterally and posteriorly. Striae moderately impressed; interstriae flat. Interstriae 1 at declivity flat in males; females unknown. Apex in lateral view square in males; females unknown. **Thoracic ventrites**. Mesoventral process rounded. Sutures between mesepimera and metanepisterna raised into a carina. Metaventrite densely covered with appressed scales. **Abdomen**. Ventrites sparsely clothed with appressed scales. Apex rounded. Males with ventrite 1 depressed medially, ventrite 5 flat. Females unknown. **Male genitalia**. Figs 46–49. Hemisternites with spiculum relictum large, bulbous and strongly pigmented. Penis with apex acute, upturned; ostial region unmodified. Endophallus with small gonoporial sclerite with reduced posterior lobes. Temones 0.52 times as long as pedon. **Female genitalia**. Unknown.

#### DNA sequences.

No DNA sequences obtained.

#### Type material examined.

**Holotype**. Male (CMNZ). Specimen pinned through right elytron; entire. Labelled ‘3832 Lake Ohau Ski Field / 1600–1650 m Johns, PM; / Nicholls, D 15.i.04’ [printed, cream card], ‘2007.215.2060’ [printed, white card], ‘*Irenimus* taxonomy / and systematics / SDJ Brown / PhD Thesis 2012–2015 / IRE4031’ [printed, cream card], ‘HOLOTYPE / *Austromonticola* / *caelibatus* / Brown 2017’ [printed, red card].

**Paratypes**. A total of 4 specimens (4 males) designated as paratypes, bearing blue paratype label. Paratype specimens deposited in CMNZ.

**MK**: Lake Ohau Ski Field [44°13.44'S, 169°46.59'E, A], 15 Jan 2004, Johns PM, Nicholls D, 1600-1650 m (CMNZ: 4).

#### Distribution.

Fig. 115. South Island: **MK**: Lake Ohau Ski Field.

#### Elevational range.

Label data: 1625 m (*n* = 5). Georeferenced data: 1574 m (*n* = 5).

#### Etymology.

From the Latin noun *caelibatus*, ‘celibacy’, an allusion to the fact that the species is thus far known only from the male sex; the species name is a noun.

#### Biology.

No plant associations recorded.

### Austromonticola furcatus

Taxon classificationAnimaliaColeopteraCurculionidae

Brown sp. n.

http://zoobank.org/0B3B6FC4-0CC0-4F66-8D01-C54894022B56

Figs 5 , 6 , 21 , 22 , 37 , 50 , 51 , 52 , 53 , 54 , 55 , 56 , 57 , 102 , 103 , 116

#### Diagnosis.

Body size medium, 4 mm in length. Dense pappolepidia on venter. Elytral declivity in females with sutural tubercle; margin of ventrite 4 produced into a lamina, with deep median emargination (Fig. 102); margin of ventrite 5 with a broad horn on either side of the genital opening (Fig. 102).

#### Description.

Body length 3.67 mm to 4.22 mm (*X*‒ = 3.98 mm, *s* = 0.19, *n* = 8). Integument black. Dorsum densely covered with brownish black to dark greyish yellowish brown appressed scales; pale “V” on elytral declivity largely confined to summits of protuberances; other pale variegations usually present on elytra, not forming patterns. Scutellum densely covered with pale scales. Pronotum same colour as elytra; with striking, pale, posterolateral maculae corresponding to pale maculae on humeral angles, especially in females. Femora and tibiae with appressed scales dense, unicolorous, concolorous with elytral scales. Tarsi integument dark reddish orange to black. **Rostrum**. Length 0.77 mm to 1.04 mm (*X*‒ = 0.90 mm, *s* = 0.09, *n* = 7), width 0.52 mm to 0.66 mm (*X*‒ = 0.57 mm, *s* = 0.05, *n* = 7), length/width ratio 1.35 to 1.68 (*X*‒ = 1.57, *s* = 0.11, *n* = 7). Epifrons with appressed scales imbricate; setae dolabriform, decumbent, concolorous; median and lateral carinae not evident. Dorsal carina arched over antennal insertions. Lateral area ventral of antennal insertions with fine setae, without appressed scales. **Antennae**. Scapes in repose reaching hind margin of eyes; covered with appressed scales and setae. Funicular segments moderately articulated; segments 1 clavate, about 1.25 times longer than 2; segments 2 clavate, about 1.4 times longer than 3; segments 3 to 4 subspherical; segments 5 to 7 oblately spheroid, subequal in length. **Pronotum**. Length 1.06 mm to 1.24 mm (*X*‒ = 1.14 mm, *s* = 0.06, *n* = 7), width 1.61 mm to 2.16 mm (*X*‒ = 1.88 mm, *s* = 0.18, *n* = 7), length/width ratio 0.80 to 0.92 (*X*‒ = 0.86, *s* = 0.04, *n* = 7); in dorsal view widest in anterior 1/4, lateral margins strongly curved anteriorly, tapering posteriorly, more pronounced in females. Anterior margin slightly emarginate medially, posterior margin straight. Disc in dorsal view evenly curved; appressed scales imbricate; setae dolabriform, decumbent, dark to concolorous. Postocular lobes poorly developed. **Elytra**. Length 2.54 mm to 3.28 mm (*X*‒ = 2.78 mm, *s* = 0.25, *n* = 7), width 1.61 mm to 2.16 mm (*X*‒ = 1.88 mm, *s* = 0.18, *n* = 7), length/width ratio 1.39 to 1.58 (*X*‒ = 1.48, *s* = 0.07, *n* = 7). Anterior margin nearly straight, humeral angles rounded. Appressed scales tessellate to narrowly imbricate. Setae claviform, decumbent, pale to concolorous. Striae moderately impressed; interstriae flat on disc, convex on elytral declivity. Interstriae 1 at declivity flat in males, produced into a strong tubercle in females. Interstriae 3 and 5 at declivity swollen in both sexes. Apex in lateral view square in males; produced ventrad in females. **Thoracic ventrites**. Mesoventral process rounded. Metaventrite densely covered with pappolepidia. **Abdomen**. Ventrites clothed almost exclusively with pappolepidia, ventrites 1 and 2 moderately densely clothed, ventrites 3 to 5 increasingly sparse. Apex rounded. Males with ventrite 1 strongly depressed medially; ventrite 5 flat. Females with ventrite 1 flat; ventrite 4 with posterior margin produced into a subtriangular lamina with very deep apical emargination (Figs 102, 103); ventrite 5 disc with median furrow, posterior margin with narrow emargination with a horn either side of emargination. **Male genitalia**. Figs 50–53. Hemisternites with spiculum relictum slender. Penis with apex acute, ostial region thickened, forming a crest. Endophallus with gonoporial sclerite having pronounced anterior lobes, lacking posterior lobes. Temones 0.75 times as long as pedon. **Female genitalia**. Figs 54–57. Distal gonocoxites slender, 2.6 times longer than high. Bursa copulatrix long; not constricted anterior of proximal gonocoxite; sclerite horseshoe-shaped, long. Sternite 8 narrowly rounded at apex, membranous laterally. Everted ovipositor 2.14 mm in length, 0.57 times body length.

#### DNA sequences. COI.

KX191344. **28S**. KX191914. **ArgK**. KX191626. **CAD**. KX191085.

#### Type material examined.

**Holotype**. Female (NZAC). Specimen mounted on card teardrop; abdomen removed, dissected and mounted in DMHF on white card below specimen; otherwise entire; elytra parted at apex. Labelled ‘NEW ZEALAND CO / Obelisk Range / Old Man Range / 14 Jan 2014/ SDJ Brown’ [printed, cream card], ‘On *Phyllachne* cushion / 1640 m / 45.3113°S 169.1956°E’ [printed, cream card], ‘*Irenimus* taxonomy / and systematics / SDJ Brown / PhD Thesis 2012–2015 / IRE4771’ [printed, cream card], ‘HOLOTYPE / *Austromonticola* / *furcatus* / Brown 2017’ [printed, red card]. Genomic DNA extract from enzyme digestion of abdomen: E196 (NZAC). CAD sequence KX191085; COI sequence KX191344; ArgK sequence KX191626; 28S sequence KX191914.

**Paratypes**. A total of 16 specimens (8 males, 8 females) designated as paratypes, bearing blue paratype label. Paratype specimens deposited in NHM, LUNZ, NZAC.

**CO**: Hyde Rock [45°23.358'S, 169°11.844'E, A], 15 Mar 1975, Watt JC, 1524 m, Litter (LUNZ: 1, NZAC: 1); Hyde Rock [45°23.358'S, 169°11.844'E, A], 22 Feb 1974, Dugdale JS, 1555-1616 m (NZAC: 1); Old Man Range [45°20.04'S, 169°12.534'E, A], 15 Mar 1975, May BM, 1524 m, Under *Celmisia* (NZAC: 1); Old Man Range [45°20.04'S, 169°12.534'E, A], 16 Jan 1965, Kuschel G, Townsend JI, 4500 feet, *Celmisia prorepens* (NHM: 1, NZAC: 4); Old Man Range [45°20.04'S, 169°12.534'E, A], 17 Jan 1965, Kuschel G, Townsend JI, 5000 feet (NHM: 1, NZAC: 1); Old Man Range [45°20.04'S, 169°12.534'E, A], 20 Feb 1974, Dugdale JS, 1615 m, Ex *Celmisia haastii* (NZAC: 1); Old Man Range [45°20.04'S, 169°12.534'E, A], 20 Feb 1974, Dugdale JS, 1615 m, ex *Celmisia sessiflora* (LUNZ: 1, NZAC: 1); The Herrons Station [45°24.739'S, 169°12.714'E, R], 17 Jan 2004, Emberson RM, Syrett P, 1590 m, Pitfall trap by tors in fell field (LUNZ: 1).

#### Distribution.

Fig. 116. South Island: **CO**: Old Man Range.

#### Elevational range.

Label data: 1372 m to 1640 m (*X*‒ = 1509 m, *s* = 103, *n* = 16). Georeferenced data: 1583 m to 1665 m (*X*‒ = 1641 m, *s* = 19, *n* = 17).

#### Etymology.

From the Latin adjective *furcatus*, ‘divided, forked’, in reference to the form of the ventral lamina of the female; the name is an adjective.

#### Biology.

Specimens have been collected in association with *Phyllachne* cushions and *Celmisia* daisies. In particular, the largest series was associated with *C. prorepens* Petrie, 1887, but specimens have also been found with *C. haastii* Hook.f., 1864, and *C. sessiliflora* Hook.f., 1864.

### Austromonticola inflatus

Taxon classificationAnimaliaColeopteraCurculionidae

Brown sp. n.

http://zoobank.org/4389A53E-83C4-460C-90E4-30ACE7D1A523

Fig. 13 , 14 , 29 , 30 , 31 , 35 , 33 , 58 , 59 , 60 , 61 , 62 , 63 , 64 , 65 , 66 , 106 , 107 , 115

#### Diagnosis.

Body size large, 8 mm in length. Rostrum with epifrons swollen (Fig. 33). Appressed scales on pronotum and elytra with metallic reflections. Females with ventrite 5 slightly emarginate and with median furrow (Fig. 106); elytra with sutural tubercle at top of elytral declivity.

#### Description.

Body length 6.98 mm to 8.72 mm (*X*‒ = 7.94 mm, *s* = 0.64, *n* = 8). Integument black. Dorsum densely covered with fine appressed scales coloured greyish blue to dark greyish blue, often with brassy or purplish metallic reflections. Femora and tibiae with appressed scales unicolorous, concolorous with elytral scales. Tarsi with integument blackish red to dark red. **Rostrum**. Length 1.29 mm to 1.72 mm (*X*‒ = 1.60 mm, *s* = 0.15, *n* = 8), width 0.89 mm to 1.18 mm (*X*‒ = 1.05 mm, *s* = 0.09, *n* = 8), length/width ratio 1.37 to 1.64 (*X*‒ = 1.53, *s* = 0.09, *n* = 8). Epifrons swollen (Fig. 33); appressed scales imbricate; setae piliform, decumbent, concolorous; median and lateral carinae not evident. Dorsal carinae arched over antennal insertions. Lateral area ventral of antennal insertions with fine setae and appressed scales. **Antennae**. Fig. 35. Scapes in repose reaching beyond hind margin of eyes; covered with appressed scales and setae. Funicular segments loosely articulated; segments 1 clavate, 1.2 times longer than 2; segments 2 clavate, about 2 times longer than 3; segments 3 to 5 clavate, getting progressively shorter; segments 6 and 7 subspherical. **Pronotum**. Length 1.68 mm to 2.26 mm (*X*‒ = 1.96 mm, *s* = 0.21, *n* = 7), width 2.63 mm to 3.62 mm (*X*‒ = 3.17 mm, *s* = 0.34, *n* = 7), length/width ratio 0.84 to 0.94 (*X*‒ = 0.89, *s* = 0.03, *n* = 7); in dorsal view widest in anterior 1/3, lateral margins evenly curved. Anterior margin entire, posterior margin straight. Disc in dorsal view with anterolateral and mediolateral impressions usually obscure, but occasionally pronounced; appressed scales imbricate; setae piliform, decumbent, concolorous. Postocular lobes moderately developed. **Elytra**. Length 4.60 mm to 6.19 mm (*X*‒ = 5.41 mm, *s* = 0.54, *n* = 7), width 2.63 mm to 3.62 mm (*X*‒ = 3.17 mm, *s* = 0.34, *n* = 7), length/width ratio 1.66 to 1.75 (*X*‒ = 1.71, *s* = 0.04, *n* = 7). Anterior margin curved posteriad in middle, with humeral angles rounded. Appressed scales imbricate. Setae piliform, semi-erect to decumbent, pale. Elytral interstriae 1 flat in males; produced into elongate tubercle in females. Apex in lateral view square in males, produced ventrad in females. **Thoracic ventrites**. Mesoventral process truncate. Metaventrite sparsely covered with appressed scales. **Abdomen**. Ventrites sparsely clothed with appressed scales. Apex of abdomen broadly rounded. Males with ventrite 1 strongly depressed medially; ventrite 5 flat. Female with subtriangular prominence on disc of ventrite 1; ventrite 4 with posterior margin curved anteriad in middle and posterior face glabrous (Figs 106, 107); ventrite 5 with median furrow and apical notch. **Male genitalia**. Figs 58–61. Hemisternites with spiculum relictum slender. Penis with apex narrowly rounded; ostial region normally developed, not strongly thickened. Endophallus with gonoporial sclerite with broad posterior lobes. Temones 0.59 times as long as pedon. **Female genitalia**. Figs 62–66. Distal gonocoxites stout, 1.3 times longer than high. Proximal gonocoxite with rods recurved distally. Bursa copulatrix stout; constricted anterior of proximal gonocoxite; sclerite large, pear-shaped. Sternite 8 broad, rounded at apex, membranous laterally.

#### DNA sequences.

**COI.** KX191461, KX191462. **28S**. KX192043, KX192044, KX192045. **ArgK**. No sequences obtained. **CAD**. KX191187, KX191188.

#### Type material examined.

**Holotype**. Female (NZAC). Specimen pinned through right elytron; abdomen removed and mounted in DMHF on white card pinned below specimen, genitalia dissected, ventrites coated in gold for SEM; left protarsus broken at base of segment 1, right mesotarsus lacking claw segment. Labelled ‘NEW ZEALAND CO / 1680 m / Hawkdun Range / 10 Dec 2013/ SDJ Brown’ [printed, cream card], ‘On *Chionohebe* and under / stones close to / *Chionohebe* cushions / 44.788°S 169.994°E’ [printed, cream card], ‘*Irenimus* taxonomy / and systematics / SDJ Brown / PhD Thesis 2012–2015 / IRE6389’ [printed, cream card], ‘HOLOTYPE / *Austromonticola* / *inflatus* / Brown 2017’ [printed, red card]. Genomic DNA extract from enzyme digestion of abdomen: E336 (NZAC). CAD sequence KX191187; COI sequence KX191461; 28S sequence KX192043.

**Paratypes**. A total of 7 specimens (3 males, 4 females) designated as paratypes, bearing blue paratype label. Paratype specimens deposited in NHM, LUNZ, NZAC.

**CO**: Hawkdun Range [44°47.256'S, 169°59.694'E, R], 11 Dec 2013, Brown SDJ, 1730 m, On *Hectorella* cushion (NHM: 1); Hawkdun Range [44°47.28'S, 169°59.64'E, R], 10 Dec 2013, Brown SDJ, 1680 m, On *Chionohebe* and under stones close to *Chionohebe* cushions (NHM: 1, LUNZ: 2, NZAC: 1); Hawkdun Range [44°49.044'S, 169°59.922'E, R], 12 Dec 2013, Brown SDJ, 1720 m, On *Hectorella* cushion (LUNZ: 1, NZAC: 1).

#### Distribution.

Fig. 115. South Island: **CO**: Hawkdun Range.

#### Elevational range.

Label data: 1680 m to 1730 m (*X*‒ = 1696 m, *s* = 23, *n* = 8). Georeferenced data: 1684 m to 1716 m (*X*‒ = 1699 m, *s* = 11, *n* = 8).

#### Etymology.

From the Latin participle *inflatus*, ‘swollen, distended’, in reference to the convex epifrons of this species; the species name is a participle.

#### Biology.

Specimens have been collected on *Hectorella caespitosa* Hook.f., 1864 and on and beside cushions of the snow hebe group (formerly placed in *Chionohebe* B.G.Briggs & Ehrend., 1976) of *Veronica*.

### Austromonticola planulatus

Taxon classificationAnimaliaColeopteraCurculionidae

Brown sp. n.

http://zoobank.org/FDDF873C-1605-4D39-9631-AA0192D0675F

Figs 11 , 12 , 27 , 28 , 32 , 67 , 68 , 69 , 70 , 71 , 72 , 73 , 74 , 75 , 108 , 109 , 115

#### Diagnosis.

Body size large, 8 mm in length. Protibia with large denticles on ventral margin (Fig. 32). Elytral disc somewhat flattened with interstriae 3 and 5 raised along length. Females with ventrite 4 with lateral laminae (Fig. 108), ventrite 5 slightly concave medially (Fig. 108); elytra interstriae 1 at top of elytral declivity flat.

#### Description.

Body length 7.59 mm to 8.25 mm (*X*‒ = 7.92 mm, *s* = 0.47, *n* = 2). Integument black. Dorsum covered with fine appressed scales, individual scales barely distinguishable, brownish black, with areas of brownish grey at sides of pronotum and base of rostrum. Femora and tibiae with appressed scales unicolorous, concolorous with elytral scales. Tarsi with integument black to strong red. **Rostrum**. Length 1.52 mm to 1.71 mm (*X*‒ = 1.62 mm, *s* = 0.13, *n* = 2), width 0.96 mm to 0.99 mm (*X*‒ = 0.98 mm, *s* = 0.02, *n* = 2), length/width ratio 1.58 to 1.73 (*X*‒ = 1.66, *s* = 0.10, *n* = 2). Epifrons with appressed scales imbricate; setae claviform, decumbent, concolorous; median and lateral carinae distinct, lateral carinae especially so. Dorsal carinae arched over antennal insertions. Lateral area ventral of antennal insertions with fine setae and appressed scales. **Antennae**. Scapes in repose reaching beyond hind margin of eyes; covered with appressed scales and setae. Funicular segments loosely articulated; segments 1 and 2 clavate, subequal, about 2 times longer than 3; segments 3 to 6 clavate, getting progressively shorter; segment 7 subconical. **Pronotum**. Length 1.92 mm to 2.28 mm (*X*‒ = 2.10 mm, *s* = 0.25, *n* = 2), width 3.22 mm to 3.47 mm (*X*‒ = 3.35 mm, *s* = 0.18, *n* = 2), length/width ratio 0.89 to 0.93 (*X*‒ = 0.91, *s* = 0.03, *n* = 2); in dorsal view widest in anterior 1/4, lateral margins evenly curved. Anterior margin sinuous, posterior margin straight. Disc in dorsal view evenly curved, except for median furrow extending from anterior 1/4 to posterior 1/8, deepest anteriorly; appressed scales imbricate; setae piliform to claviform, decumbent, dark. Postocular lobes strongly developed. **Elytra**. Length 5.21 mm to 5.39 mm (*X*‒ = 5.30 mm, *s* = 0.13, *n* = 2), width 3.22 mm to 3.47 mm (*X*‒ = 3.35 mm, *s* = 0.18, *n* = 2), length/width ratio 1.50 to 1.67 (*X*‒ = 1.59, *s* = 0.12, *n* = 2). Anterior margin almost straight, humeral angles rounded. Disc subdepressed. Appressed scales imbricate. Setae piliform to claviform, decumbent to semi-erect, concolorous on disc, pale laterally and posteriorly. Striae strongly impressed; interstriae convex; interstriae 1 at top of elytral declivity flat in males, swollen in females; interstriae 3 and 5 raised throughout length in both sexes. Apex in lateral view square in males; slightly produced ventrad and with small subapical tubercles in females. **Thoracic ventrites**. Mesoventral process narrowly rounded. Metaventrite densely covered with appressed scales. **Abdomen**. Ventrites densely covered with appressed scales. Males with ventrite 1 flat; ventrite 5 flat. Females with ventrite 1 flat; ventrite 4 with posterior margin produced laterally into small subtriangular laminae (Figs 108, 109); ventrite 5 with median concavity. Apex rounded. **Legs**. Protibiae with conspicuous denticles on ventral margin. **Male genitalia**. Figs 67–70. Hemisternites with spiculum relictum inconspicuous, possibly absent. Penis with apex sagittate, broad; ostial region normally developed. Endophallus with papillae; gonoporial sclerite with long, thin posterior lobes. Temones 0.73 times as long as pedon. **Female genitalia**. Figs 71–75. Distal gonocoxites moderately stout, 1.9 times longer than high. Bursa copulatrix stout, not constricted anterior of proximal gonocoxite; sclerite horseshoe-shaped. Sternite 8 fully sclerotised, apex rounded.

#### DNA sequences.

No DNA sequences obtained.

#### Type material examined.

**Holotype**. Female (NZAC). Specimen pinned through right elytron; abdomen removed and mounted in DMHF on white card pinned below specimen, genitalia dissected, ventrites coated in gold for SEM; otherwise entire. Labelled ‘Mt Bitterness / St Mary Range CO:NZ / 1830–1900 m / P.M.Johns & / M.H.Ingerfeld / 6–7.II.78’ [printed, white card], ‘stonefield with / occ. mat plants’ [printed, white card], ‘*Irenimus* taxonomy / and systematics / SDJ Brown / PhD Thesis 2012–2015 / IRE7625’ [printed, cream card], ‘HOLOTYPE / *Austromonticola* / *planulatus* / Brown 2017’ [printed, red card].

**Paratypes**. A total of 1 specimen (1 male) designated as paratype, bearing blue paratype label. Paratype specimen deposited in CMNZ.

**CO**: Mt Bitterness [44°45.24'S, 170°18.198'E, A], 6–7 Feb 1978, Johns PM, Ingerfeld MH, 1830-1900 m, Stonefield with occasional mat plants (CMNZ: 1).

#### Distribution.

Fig. 115. South Island: **CO**: St Marys Range.

#### Elevational range.

Label data: 1865 m (*n* = 2).Georeferenced data: 1905 m (*n* = 2).

#### Etymology.

From the Latin adjective *planus*, ‘flat, even’, combined with the the diminutive -*ulus* and the possessive -*atus*, referring to the almost level dorsum of this species, as compared with others in the genus; the species name is an adjective.

#### Biology.

Collected in fellfield. No plant associations recorded.

### Austromonticola postinventus

Taxon classificationAnimaliaColeopteraCurculionidae

Brown sp. n.

http://zoobank.org/0905AF10-6CBD-4E24-B7F2-24D099931F84

Figs 9 , 10 , 25 , 26 , 33 , 36 , 76 , 77 , 78 , 79 , 80 , 81 , 82 , 83 , 84 , 110 , 111 , 115

#### Diagnosis.

Body size large, 8 mm in length. Epifrons flat, with decumbent setae. Females with ventrite 5 emarginate and with median swelling (Fig. 110); elytra with interstriae 1 swollen at top of elytral declivity and the apex produced ventrad.

#### Description.

Body length 7.26 mm to 8.42 mm (*X*‒ = 7.84 mm, *s* = 0.82, *n* = 2). Integument black. Dorsum densely covered with fine brownish black appressed scales with purple and gold metallic reflectance, reflectance particularly pronounced laterally and posteriorly. Femora and tibiae with appressed scales dense, unicolorous, concolorous with elytral scales. Tarsi with integument strong red. **Rostrum**. Length 1.45 mm to 1.67 mm (*X*‒ = 1.56 mm, *s* = 0.16, *n* = 2), width 0.90 mm to 1.02 mm (*X*‒= 0.96 mm, *s* = 0.08, *n* = 2), length/width ratio 1.61 to 1.64 (*X*‒ = 1.62, *s* = 0.02, *n* = 2). Epifrons with appressed scales tessellate; setae piliform, decumbent, pale; median and lateral carinae not evident. Dorsal carinae arched over antennal insertions. Lateral area ventral of antennal insertions with fine setae and appressed scales. **Antennae**. Fig. 36. Scapes in repose reaching hind margin of eyes; covered with appressed scales and setae. Funicular segments moderately articulated; segments 1 clavate, about 1.3 times longer than 2; segments 2 clavate, about 2 times longer than 3; segments 3 clavate, slightly longer than 4; segments 4 to 6 clavate, subequal; segments 7 subconical. **Pronotum**. Length 1.90 mm to 2.11 mm (*X*‒ = 2.00 mm, *s* = 0.15, *n* = 2), width 2.85 mm to 3.53 mm (*X*‒ = 3.19 mm, *s* = 0.48, *n* = 2), length/width ratio 0.85 to 0.90 (*X*‒ = 0.88, *s* = 0.04, *n* = 2); in dorsal view widest in anterior 1/3, lateral margins evenly curved. Anterior margin entire, posterior margin straight. Disc in dorsal view evenly curved; appressed scales imbricate; setae piliform, decumbent, concolorous. Postocular lobes moderately developed. **Elytra**. Length 4.90 mm to 5.70 mm (*X*‒ = 5.30 mm, *s* = 0.57, *n* = 2), width 2.85 mm to 3.53 mm (*X*‒ = 3.19 mm, *s* = 0.48, *n* = 2), length/width ratio 1.61 to 1.72 (*X*‒ = 1.67, *s* = 0.07, *n* = 2). Anterior margin almost straight, humeral angles rounded. Appressed scales imbricate. Setae piliform, erect, pale. Striae moderately impressed; interstriae flat; interstriae 1 at elytral declivity flat in males, with small tubercle in females. Apex in lateral view square in males, produced posteriad in females. **Thoracic ventrites**. Mesoventral process acutely rounded in males, broadly rounded in females. Metaventrite densely covered with appressed scales. **Abdomen**. Ventrites densely covered with appressed scales. Apex rounded. Males with ventrite 1 depressed medially; ventrite 5 flat. Females with ventrite 1 flat; ventrite 4 posterior margin curved anteriad in middle, with narrow laminae laterally (Figs 110, 111); ventrite 5 swollen medially, posterior margin broadly emarginate. **Male genitalia**. Figs 76–79. Hemisternites with spiculum relictum slender. Penis with apex narrowly rounded, upturned; ostial region normally developed. Endophallus with gonoporial sclerite small, posterior lobes reduced. Temones 0.63 times as long as pedon. **Female genitalia**. Figs 80–84. Distal gonocoxites stout, 1.4 times longer than high. Bursa copulatrix stout; constricted anterior of proximal gonocoxite; sclerite large, pear-shaped. Sternite 8 broad, rounded at apex, membranous laterally.

#### DNA sequences.

No DNA sequences obtained.

#### Type material examined.

**Holotype**. Female (NZAC). Specimen pinned through right elytron; abdomen removed and mounted in DMHF on white card pinned below specimen, genitalia dissected, ventrites coated in gold for SEM; right proleg missing, broken off from trochanter. Labelled ‘Mt Kirkliston / 6000 / C.J.Burrows / 5.1.65 / understones’ [first three lines printed, last two lines handwritten, off-white card], ‘*Irenimus* taxonomy / and systematics / SDJ Brown / PhD Thesis 2012–2015 / IRE1116’ [printed, off-white card], ‘HOLOTYPE / *Austromonticola* / *postinventus* / Brown 2017’ [printed, red card].

**Paratypes**. A total of 1 specimen (1 male) designated as paratype, bearing blue paratype label. Paratype specimen deposited in NZAC.

**SC**: Kirkliston Range [44°32.124'S, 170°30.954'E, A], 8–9 Feb 1978, Johns PM, Ingerfeld MH, 1740-1770 m, Stonefield with occasional mat plants (NZAC: 1).

#### Distribution.

Fig. 115. South Island: **SC**: Kirkliston Range.

#### Elevational range.

Label data: 1755 m to 1829 m (*n* = 2). Georeferenced data: 1615 m to 1868 m (*n* = 2).

#### Etymology.

From the Latin prefix *post*, ‘after’, and the participle *inventus*, ‘discovered’, referring to the recognition of this species after my PhD defence; the name is a participle.

#### Biology.

Collected in fellfield. No plant associations recorded.

### Austromonticola mataura

Taxon classificationAnimaliaColeopteraCurculionidae

Brown sp. n.

http://zoobank.org/8F3D76BE-96EF-4C84-8833-54ED30B20938

Figs 3 , 4 , 19 , 20 , 85 , 86 , 87 , 88 , 89 , 90 , 91 , 92 , 104 , 105 , 116

#### Diagnosis.

Body size medium, 4 mm in length. Venter with glossy appressed scales, pappolepidia sparse. Elytral declivity of females with sutural tubercle; margin of ventrite 3 with paired median processes; margin of ventrite 4 produced into a lamina, with deep median emargination; margin of ventrite 5 with a broad horn on either side of the genital opening.

#### Description.

Body length 3.42 mm to 4.11 mm (*X*‒ = 3.80 mm, *s* = 0.25, *n* = 13). Integument black. Dorsum densely covered with light bluish grey to dark greyish yellow appressed scales with metallic reflections; frequently with pale mottling sublaterally, and with scutellum, humeral area and hind pronotal angles bluish white; elytral declivity slightly paler than disc, especially in males. Pappolepidia of mesothoracic sternites light yellow. Femora appressed scales concolorous with elytral scales, often with obscure pale band in distal 1/4. Tarsi with integument deep orange to strong red. **Rostrum**. Length 0.72 mm to 0.88 mm (*X*‒ = 0.84 mm, *s* = 0.06, *n* = 8), width 0.52 mm to 0.66 mm (*X*‒ = 0.57 mm, *s* = 0.05, *n* = 7), length/width ratio 1.35 to 1.68 (*X*‒ = 1.57, *s* = 0.11, *n* = 7). Epifrons with appressed scales imbricate; setae claviform, decumbent, concolorous; median carina weak; lateral carinae evident. Dorsal carinae arched over antennal insertions. Lateral area ventral of antennal insertions with fine setae, without appressed scales. **Antennae**. Scapes in repose reaching middle of eyes. Funicular segments moderately articulated; segments 1 and 2 clavate, subequal, about 2 times longer than 3; segments 3 and 4 clavate, subequal; segments 5 to 7 subspherical, subequal. **Pronotum**. Length 1.06 mm to 1.18 mm (*X*‒ = 1.13 mm, *s* = 0.04, *n* = 8), width 1.61 mm to 2.16 mm (*X*‒ = 1.88 mm, *s* = 0.18, *n* = 7), length/width ratio 0.80 to 0.92 (*X*‒ = 0.86, *s* = 0.04, *n* = 7); in dorsal view widest in anterior 1/3, lateral margins strongly curved to widest point, gently curved behind. Anterior margin entire, posterior margin straight. Disc in dorsal view uneven, weak median furrow present, anterolateral depressions vague; appressed scales tessellate to imbricate; setae claviform, decumbent to semi-erect, concolorous. Postocular lobes poorly developed. **Elytra**. Length 2.30 mm to 3.04 mm (*X*‒ = 2.79 mm, *s* = 0.23, *n* = 8), width 1.61 mm to 2.16 mm (*X*‒ = 1.88 mm, *s* = 0.18, *n* = 7), length/width ratio 1.39 to 1.58 (*X*‒ = 1.48, *s* = 0.07, *n* = 7). Anterior margin curved posteriad in middle, humeral angles rounded. Appressed scales tessellate to imbricate. Setae claviform, decumbent to semi-erect, concolorous. Interstriae 1 at elytral declivity flat in males, produced into tubercles in females. Interstriae 3 raised at base; swollen at elytral declivity in both sexes, though more pronounced in females. Interstriae 5 at elytral declivity swollen in both sexes. Humeral region strongly pronounced by deeply impressed striae 9. Apex in lateral view square in males, produced ventrad in females. **Thoracic ventrites**. Mesoventral process narrowly rounded. Mesanepisterna, mesepimera and metanepisterna covered with small pappolepidia, contrasting with metaventrite densely covered with appressed scales. **Abdomen**. Ventrites clothed almost exclusively with appressed scales; ventrites 1 and 2 densely clothed in females, scales dense laterally and sparser medially in males; ventrites 3 to 5 increasingly sparse. Males with ventrite 1 depressed medially; ventrite 5 flat. Females with ventrite 1 flat; ventrite 4 posterior margin produced into a lamina, usually with a deep median emargination (Fig. 104, 105) but variably shallower to entire; ventrite 5 disc with median furrow, deeply emarginate with a broad horn on either side of emargination. Apex narrowly rounded. **Male genitalia**. Fig. 85–88. Hemisternites with spiculum relictum slender. Penis with apex acute, upturned; ostial region thickened, forming a crest. Endophallus with gonoporial sclerite small, anterior and posterior lobes reduced. Temones 0.71 times as long as pedon. **Female genitalia**. Fig. 89–92. Distal gonocoxites slender, 3.1 times longer than high. Proximal gonocoxite with rods recurved distally. Bursa copulatrix long; not constricted anterior of proximal gonocoxite; sclerite small, semicircular. Sternite 8 narrow, apex acute, membranous laterally.

#### DNA sequences.

**COI.** KX191347, KX191348, KX191349. **28S**. KX191917, KX191918, KX191919. **ArgK**. KX191629, KX191630, KX191631. **CAD**. KX191088, KX191089, KX191090.

#### Type material examined.

**Holotype**. Female (NZAC). Specimen mounted on card teardrop; abdomen removed, dissected and mounted in DMHF on white card below specimen; otherwise entire. Labelled ‘NEW ZEALAND OL / Mt Dick / Kingston / 17 Jan 2014 / SDJ Brown’ [printed, cream card], ‘On *Phyllachne* cushion / 1690 m / 45.2652°S 168.6870°E’ [printed, cream card], ‘*Irenimus* taxonomy / and systematics / SDJ Brown / PhD Thesis 2012–2015 / IRE4775’ [printed, cream card], ‘HOLOTYPE / *Austromonticola* / *mataura* / Brown 2017’ [printed, red card]. Genomic DNA extract from enzyme digestion of abdomen: E200 (NZAC). CAD sequence KX191089; COI sequence KX191348; ArgK sequence KX191630; 28S sequence KX191918.

**Paratypes**. A total of 21 specimens (9 males, 12 females) designated as paratypes, bearing blue paratype label. Paratype specimens deposited in AMNZ, ANIC, NHM, CMNZ, IACC, LUNZ, MONZ, NZAC, USNM.

**OL**: Mt Dick [45°15.564'S, 168°40.926'E, R], 17 Jan 2014, Brown SDJ, 1600 m, On *Dracophyllum muscoides* cushion (AMNZ: 1, LUNZ: 2, MONZ: 2, NZAC: 1); Mt Dick [45°15.696'S, 168°41.016'E, R], 17 Jan 2014, Brown SDJ, 1680 m, On *Phyllachne* cushion (NHM: 1, USNM: 1); Mt Dick [45°15.81'S, 168°41.148'E, R], 17 Jan 2014, Brown SDJ, 1710 m, On *Raoulia haastii* (LUNZ: 1, USNM: 1); Mt Dick [45°17.112'S, 168°41.19'E, R], 16 Jan 2014, Brown SDJ, 1570 m, On *Phyllachne* cushion (ANIC: 1); Mt Dick [45°18.264'S, 168°41.496'E, R], 16 Jan 2014, Brown SDJ, 1510 m, On *Phyllachne* cushion (AMNZ: 1, ANIC: 1, NHM: 1, CMNZ: 1, LUNZ: 1, NZAC: 1); Symmetry Peaks [45°16.928'S, 168°34.56'E, A], 8 Jan 1987, Barratt BIP, 1750-1860 m (IACC: 1); Upper Mataura Valley [45°19.734'S, 168°26.07'E, A], 17 Jan 1971, 1524 m, Moss (NZAC: 3).

#### Distribution.

Fig. 116. South Island: **OL**: Eyre Mountains; Mt Dick.

#### Elevational range.

Label data: 1270 m to 1805 m (*X*‒ = 1580 m, *s* = 107, *n* = 23). Georeferenced data: 878 m to 1682 m (*X*‒ = 1479 m, *s* = 254, *n* = 23).

#### Etymology.

This species is named after its distribution in the headwaters of the Mataura River; the name is a noun in apposition. The word mataura is Māori, of obscure meaning. Mataura was an ancestor of Ngatoro-i-rangi, the priest of the Arawa canoe. It may mean `glowing face', which is appropriate for its collection localities thus far have been on the eastern slopes of the Eyre Mountains.

#### Biology.

Collected from *Raoulia hectorii* Hook.f., 1864, (recorded as *R. haastii* Hook.f., 1864, in error), moss, *Dracophyllum muscoides* Hook.f., 1864, and *Phyllachne* cushions. The majority of specimens were collected from *Phyllachne*.

### Austromonticola rotundus

Taxon classificationAnimaliaColeopteraCurculionidae

Brown sp. n.

http://zoobank.org/3B99FAB1-E825-4593-8508-4411388A6D40

Figs 7 , 8 , 23 , 24 , 38 , 93 , 94 , 95 , 96 , 97 , 98 , 99 , 112 , 113 , 117

#### Diagnosis.

Body size medium, 4.5 mm in length. Pronotum with subparallel lateral margins (Fig. 38), about as wide as base of elytra. Venter clothed with appressed scales, pappolepidia sparse. Females with elytral declivity distinctly rounded, without sutural tubercle; margin of ventrite 5 entire (Fig. 112).

#### Description.

Body length 4.20 mm to 4.80 mm (*X*‒ = 4.50 mm, *s* = 0.20, *n* = 11). Integument black. Dorsum densely covered with moderate olive to greyish brown appressed scales, some variegation usually present on elytra, but rarely forming distinct patterns; pronotum frequently with obscure lighter lines obliquely converging anteriorly. Femora and tibiae with dense appressed scales concolorous with elytral scales, usually with pale band in distal 1/4 of femur. Tarsi with integument deep orange. **Rostrum**. Length 0.89 mm to 0.99 mm (*X*‒ = 0.94 mm, *s* = 0.04, *n* = 6), width 0.58 mm to 0.66 mm (*X*‒ = 0.62 mm, *s* = 0.03, *n* = 6), length/width ratio 1.44 to 1.69 (*X*‒ = 1.52, *s* = 0.09, *n* = 6). Epifrons with appressed scales imbricate; setae claviform, decumbent, concolorous; median and lateral carinae not evident. Dorsal carinae arched over antennal insertions. Lateral area ventral of antennal insertions with fine setae and with appressed scales. **Antennae**. Scapes in repose reaching hind margin of eyes; covered with appressed scales and setae. Funicular segments moderately articulated; segments 1 clavate, about 1.5 times longer than 2; segments 2 clavate, about 1.2 times longer than 3; segments 3 and 4 clavate; segments 5 to 7 subspherical, subequal in length. **Pronotum**. Length 1.26 mm to 1.39 mm (*X*‒ = 1.32 mm, *s* = 0.05, *n* = 6), width 1.79 mm to 2.26 mm (*X*‒ = 1.98 mm, *s* = 0.19, *n* = 6), length/width ratio 0.83 to 0.91 (*X*‒ = 0.87, *s* = 0.03, *n* = 6); in dorsal view widest in anterior 1/3, lateral margins approximately subparallel (Fig. 38). Anterior margin entire, posterior margin curved. Disc in dorsal view evenly curved, but with obscure median depression in anterior 1/3; appressed scales imbricate; setae claviform, decumbent, concolorous. Postocular lobes poorly developed. **Elytra**. Length 2.79 mm to 3.52 mm (*X*‒ = 3.02 mm, *s* = 0.26, *n* = 6), width 1.79 mm to 2.26 mm (*X*‒ = 1.98 mm, *s* = 0.19, *n* = 6), length/width ratio 1.43 to 1.62 (*X*‒ = 1.53, *s* = 0.07, *n* = 6). Anterior margin curved posteriad, humeral angles rounded. Appressed scales imbricate. Setae claviform, semi-erect, pale. Striae moderately impressed; interstriae slightly convex. Interstriae 1 at declivity flat in both sexes. Interstriae 3 at declivity flat in both sexes. Elytral declivity curved in females. Apex in lateral view square in males; produced ventrad in females. **Thoracic ventrites**. Mesoventral process rounded. Mesanepisterna, mesepimera, metanepisterna and metaventrite densely covered with appressed scales. **Abdomen**. Ventrites sparsely clothed with appressed scales. Apex broadly rounded. Males with ventrite 1 flat; ventrite 5 flat. Females with ventrite 1 flat; ventrite 4 with posterior margin produced into a broad lamina with a strong apical emargination (Figs 112, 113); ventrite 5 disc with shallowly concave, posterior margin entire. **Male genitalia**. Figs 93, 94. Penis with apex sagittate; ostial region receeding anteriorly, not thickened. Endophallus with gonoporial sclerite very small. Temones 0.78 times as long as pedon. **Female genitalia**. Figs 95–99. Distal gonocoxites slender, 3.0 times longer than high. Bursa copulatrix long; not constricted anterior of proximal gonocoxite; sclerite lanceolate. Sternite 8 apex rounded, fully sclerotised.

#### DNA sequences.

**COI.** KX191445. **28S**. KX192022. **ArgK**. KX191729. **CAD**. KX191173.

#### Type material examined.

**Holotype**. Female (NZAC). Specimen mounted on card teardrop; abdomen removed, dissected and mounted in DMHF on white card below specimen; otherwise entire. Labelled ‘NEW ZEALAND CO / Obelisk Range / Old Man Range / 13 Jan 2014 / SDJ Brown’ [printed, cream card], ‘On *Dracophyllum* / *muscoides* cushion / 1590 m / 45.3126°S 169.2102°E’ [printed, cream card], ‘*Irenimus* taxonomy / and systematics / SDJ Brown / PhD Thesis 2012–2015 / IRE4888’ [printed, cream card], ‘HOLOTYPE / *Austromonticola* / *atriarius* / Brown 2017’ [printed, red card]. Genomic DNA extract from enzyme digestion of abdomen: E313 (NZAC). CAD sequence KX191173; COI sequence KX191445; ArgK sequence KX191729; 28S sequence KX192022.

**Paratypes**. A total of 17 specimens (6 males, 11 females) designated as paratypes, bearing blue paratype label. Paratype specimens deposited in NHM, IACC, LUNZ: 1, NZAC: 2.

**CO**: North Garvie Mountains, 9 Feb 1985, Barratt BIP, 1200 m, Ex *Geum parviflorum* (IACC: 1); Old Man Range [45°20.04'S, 169°12.534'E, A], 17 Jan 1965, Kuschel G, Townsend JI, 5000 feet (NZAC: 1); Old Woman Range [45°15.18'S, 169°3.54'E, A], 20 Nov 1974, Watt JC, 1389 m, Litter (LUNZ: 1, NZAC: 5); Rock Peak [44°59.442'S, 168°58.17'E, A], 27 Nov 1974, Dugdale JS, 1430-1460 m, Litter (NZAC: 1); Rock Peak [44°59.442'S, 168°58.17'E, A], 27 Nov 1974, Dugdale JS, 1430-1460 m, Mixed swards and litter (NHM: 2, LUNZ: 1, NZAC: 4); Rock Peak [44°59.442'S, 168°58.17'E, A], 27 Nov 1974, Dugdale JS, 1430-1460 m, Mixed swards litter (NZAC: 1).

#### Distribution.

Fig. 117. South Island: **CO**: Garvie Mountains; Old Man Range; Old Woman Range; Crown Range.

#### Elevational range.

Label data: 1200 m to 1590 m (*X*‒ = 1428 m, *s* = 76, *n* = 18). Georeferenced data: 1329 m to 1717 m (*X*‒ = 1477 m, *s* = 184, *n* = 17).

#### Etymology.

From the Latin adjective *rotundus*, ‘round, spherical’ for the form of the female elytral declivity; the name is an adjective.

#### Biology.

Specimens have been collected from *Geum parviflorum* Smith, 1805 and *Dracophyllum muscoides*. The majority of specimens, however, were collected from litter and turf (sward) samples.

### Key to the species of *Austromonticola*

**Table:** 

1	Larger species, greater than 7 mm in length	**2**
–	Smaller species, less than 5 mm in length	**5**
2(1)	Denticles on protibiae large, conspicuous (Fig. 32); lateral carinae of rostrum distinct; interstriae 3 and 5 raised along length	***A. planulatus***
–	Denticles on protibiae undeveloped (Fig. 31); lateral carinae of rostrum moderate or weak; interstriae 3 and 5 raised at base and/or on elytral declivity, not raised on disc	**3**
3(2)	Epifrons swollen, convex (Fig. 33). Funicle segments 7 subspherical (Fig. 35)	***A. inflatus***
–	Epifrons flattened, level (Fig. 34). Funicle segments 7 subconical (Fig. 36)	**4**
4(3)	Epifrons with setae semi-erect. Setae along elytral interstriae 7 erect	***A. caelibatus***
–	Epifrons with setae decumbent. Setae along elytral interstriae 7 decumbent	***A. postinventus***
5(1)	Pronotum hexagonal in outline, widest anteriorly, sides evenly converging toward base (Fig. 37). Females with elytral declivity roughly vertical, sutural tubercle present; ventrite 5 emarginate, possessing spines around the genital opening	**6**
–	Pronotum round in outline (Fig. 38). Female with elytral declivity rounded, without sutural tubercle; margin of ventrite 5 entire	***A. rotundus***
6(5)	Venter with dense pappolepidia, round appressed scales sparsely distributed	**7**
–	Venter with dense appressed scales, pappolepidia sparsely distributed	***A. mataura***
7(6)	Pronotum with median furrow. Elongate elytral scales decumbent. Antennal funicle segments 3 longer than 4	***A. atriarius***
–	Pronotum evenly curved. Elongate elytral scales semi-erect. Antennal funicle segments 3 of similar length as 4	***A. furcatus***

### Molecular diagnostics

Specimens of five species of *Austromonticola* were available for DNA sequencing. No fresh specimens of *A. planulatus*, *A. caelibatus* and *A. postinventus* were collected. Multiple specimens were available only of *A. inflatus* and *A. mataura*, and only the latter yielded multiple sequences for all gene regions. Due to these low sample numbers, conclusions regarding intra-specific variability are necessarily limited.

The three protein-coding genes could all be unambiguously aligned, 28S being the only locus that required alignment gaps. The COI alignment was divided into two regions. The first represented the 5' region, corresponding to the region favoured for DNA barcoding (Hebert et al. 2003), and consisted of 669 bp, beginning at position 1239 of the *Tribolium castaneum* (Herbst, 1797) mitochondrial genome KM244661.1. This region was only sequenced for *A. mataura* and *A. rotundus* due to difficulties in amplifying it in other species. The second region, at the 3' end of the gene, consisted of 799 bp beginning at position 1909 of the same *T. castaneum* mitochondrial genome sequence. The 28S alignment was 756 bp long, beginning at position 1121 of the *Tenebrio* sp. reference sequence AY210843.1 (Gillespie et al. 2004). The ArgK alignment was 681 bp long, beginning at position 419 of the *T. castaneum* reference sequence XM_966707.4. The CAD alignment was 460 bp long, beginning at position 2082 of the *T. castaneum* reference sequence XM_967097.3.

Genetic variation existed in all gene regions, COI showing the greatest amount of variation, followed by CAD and ArgK, and 28S displaying the least (Figs 122–124). Of the genes sampled here, COI exhibited the greatest amount of genetic variation, as expected (Lin and Danforth 2004).

COI proved to be the most suitable gene for identifying specimens of *Austromonticola*. The 3' end of COI allowed unambiguous differentiation of all species with available data. This region has the greatest taxon coverage, though indications are that the 5' ‘barcoding’ end of the gene has higher levels of variation if amplification is successful (Fig. 122). ArgK is also a possible candidate for identification purposes, as all species displayed differences between them, however the level of variation was substantially lower than that of COI (Fig. 123). 28S and CAD are both unsuitable for specimen identification, due to there being some zero distances between species (Figs 124, 121).

The same pattern of variation in each gene region was observed when the number of diagnostic nucleotides was calculated (Figs 125–129). All species can be diagnosed using COI, with the number of nucleotides ranging between 7 and 20, and a median of 15. However, due to the lack of intra-specific sampling, these diagnostic sites should be used with caution.

Across all three protein-coding genes, *A. atriarius*, *A. furcatus* and *A. mataura* showed the smallest interspecific distances. In COI, *A. rotundus* was nearest to *A. inflatus* (Fig. 120), while in CAD and ArgK it was nearest to *A. atriarius* (Fig. 121, 123) and in 28S it was nearest to *A. mataura* (Fig. 124). There were no differences in the 28S sequences of *A. atriarius*, *A. furcatus* and *A. inflatus* (Fig. 124).

### Phylogenetic analysis

Analysis of the character matrix (Table 2) resulted in a single most parsimonious cladogram (Fig. 118), with a length of 68 steps, a consistency index of 57 and a retention index of 70. Collapsing unsupported nodes (Fig. 119) increased the tree length to 78 steps.

**Table 2.** Character matrix for cladistic analysis of relationships within *Austromonticola*.

Taxon	1	6	11	16	21	26	31
*Austromonticola atriarius*	02100	00100	11000	11011	10010	01101	11?
*Austromonticola caelibatus*	12101	00010	00001	1?21?	0????	???01	021
*Austromonticola furcatus*	02101	00100	11000	01011	10010	11101	111
*Austromonticola inflatus*	12101	00010	00001	01210	00110	11101	021
*Austromonticola planulatus*	12100	00000	00000	10211	01010	01111	011
*Austromonticola postinventus*	12101	00010	00001	01210	00110	01101	021
*Austromonticola mataura*	02101	00100	11000	11011	10010	11101	111
*Austromonticola rotundus*	02101	00100	00000	10011	00010	01111	01?
*Irenimus parilis*	00110	00001	00000	10000	01021	00020	000
*Brachyolus punctatus*	00010	01000	00000	00100	01000	00000	010
*Inophloeus sulcifer*	11001	01000	01110	00100	01011	00001	011
*Zenagraphus metallescens*	11000	11000	00110	00000	01011	02100	010
*Inophloeus sternalis*	10000	01001	00000	10100	00021	00001	021
Undescribed genus and species	11000	10000	1001?	00100	00010	00120	021

In the discussion of characters below, the significance of synapomorphies is only discussed in relation to *Austromonticola*, due to limited representation of outgroup taxa.

1. Body length, as measured from the anterior margin of the eyes to the elytral declivity in lateral view: (0) less than 6 mm; (1) greater than 6 mm. As estimated by this phylogeny, state 1 is the ancestral body size, while state 0 is homoplasious for the *A. rotundus*–*A. mataura* clade and *Irenimus parilis* + *Brachyolus punctatus* (ci = 0.5, ri = 0.8).

2. Body vestiture, form of appressed scales covering dorsum: (0) scales large, conspicuous, imbricate, coloured brown or bronze or pale yellow; (1) scales small, inconspicuous, tessellate, dome-shaped, black, ridges not visible at 50 × magnification; (2) scales small, tessellate, flat, coloured greys or browns, ridges visible at 30 × magnification. State 2 is a synapomorphy for *Austromonticola* (ci = 1, ri = 1).

3. Labium, form of base: (0) flat; (1) concave, with lateral areas raised relative to disc. State 1 is a synapomorphy for *Austromonticola* but convergently present in *Irenimus parilis* (ci = 0.5, ri = 0.75).

4. Labium, setation of disc: (0) bare; (1) with setae distally and laterally. The plesiomorphic state 0 is present in all species of *Austromonticola*, with state 1 being a synapomorphy for *Irenimus parilis* + *Brachyolus punctatus* (ci = 1, ri = 1).

5. Rostrum, ventral side, hypostomal-labial sutures: (0) strongly convergent to point distal of head capsule deflexion; (1) roughly parallel, converging to point proximal of head deflexion. State 1 is convergently present in *Inophloeus sulcifer* and *Austromonticola* excluding *A. planulatus*, though with a reversal in *A. atriarius* (ci = 0.33, ri = 0.67).

6. Frons, vestiture: (0) clothed with pale, thick, conspicuous setae; (1) clothed with inconspicuous setae. The plesiomorphic state 0 is present in all species of *Austromonticola*, with state 1 being a synapomorphy for *Zenagraphus metallescens* + the undescribed genus (ci = 1, ri = 1).

7. Pronotum, sculpture of disc: (0) evenly convex, without obvious sculpture; (1) with depressions or wrinkles laterally. State 0 is shared with the undescribed genus and *I. parilis* by all species of *Austromonticola* (ci = 0.33, ri = 0.33).

8. Pronotum, scale pattern: (0) without distinctive pattern; (1) with lateral vittae formed by pale scales, vittae extending onto humeral area. State 1 is a synapomorphy for the *A. rotundus*–*A. mataura* clade (ci = 1, ri = 1).

9. Protarsus, second segment: (0) transverse, or width subequal to length; (1) distinctly elongate, length greater than width. State 1 is a synapomorphy for the *A. caelibatus*–*A. inflatus* clade (ci = 1, ri = 1).

10. Metatibial apex: (0) simple, without corbel; (1) with bare corbel. The plesiomorphic state 0 is present in all species of *Austromonticola*. This phylogeny estimates that state 1 is convergently present in *Inophloeus sternalis* and *Irenimus parilis* (ci = 0.5, ri = 0).

11. Metanepisterna, vestiture: (0) consisting of round appressed scales; (1) consisting of pappolepidia. State 1 is a synapomorphy for the *A. atriarius*–*A. mataura* clade, but convergently present in the undescribed genus (ci = 0.5, ri = 0.67).

12. Metanepisterna, vestiture: (0) comprising two rows of scales; (1) comprising three or more rows of scales. State 1 is a synapomorphy for the *A. atriarius*–*A. mataura* clade but convergently present in *I. sulcifer* (ci = 0.5, ri = 0.67) (ci = 0.5, ri = 0.67).

13. Elytra, form of strial punctures: (0) shallow, circular, interstriae much wider than punctures; (1) deep, subquadrate, interstriae approximately equal in width as punctures. The plesiomorphic state 0 is present in all species of *Austromonticola*. State 1 is convergently present in *Inophloeus sulcifer* and *Zenagraphus metallescens* (ci = 0.5, ri = 0).

14. Elytra, length and density of setae on disc: (0) long, conspicuous and evenly distributed; (1) short, sparse. The plesiomorphic state 0 is present in all species of *Austromonticola*. State 1 is a synapomorphy for the *Inophloeus sulcifer*–*Zenagraphus metallescens* clade (ci = 1, ri = 1).

15. Elytra, form of setae: (0) claviform; (1) piliform. State 1 is a synapomorphy for the *A. caelibatus*–*A. inflatus* clade (ci = 1, ri = 1).

16. Elytra, humeral region anteriorly of conjunction of striae 7 & 8: (0) evenly convex, not prominent in comparison with surroundings, stria 9 not deeply impressed at base; (1) strongly raised in comparison with surroundings, stria 9 deeply impressed at base. A complex character that does not show any clear relationships (ci = 0.25, ri = 0.5).

17. Elytra, form of sutural interval above elytral declivity in females: (0) flat; (1) developed into a prominent tubercle. State 1 is convergently present in the *A. caelibatus*–*A. inflatus* clade and the *A. atriarius*–*A. mataura* clades (ci = 0.5, ri = 0.75). The unknown female of *A. caelibatus* is predicted by this estimation to possess state 1.

18. Elytra, form of interstriae 3 above elytral declivity: (0) swollen, more so than on disc; (1) developed into prominent tubercles; (2) flat, no greater than on disc. State 2 is shared between *A. planulatus* and the *A. caelibatus*–*A. inflatus* clade. State 0 is a shared by all members of the *A. rotundus*–*A. mataura* clade but has also evolved elsewhere in the tree (ci = 0.5, ri = 0.67).

19. Elytra, form of interstriae near apex: (0) clearly impressed, confluence of interstriae 3 and 9 clearly raised above confluence of 2 and 10; (1) striae obsolete, confluence of interstriae 3 and 9 on same level as confluence of 2 and 10. State 1 is a synapomorphy for *Austromonticola* (ci = 1, ri = 1).

20. Ventrite 4 of females, form of posterior margin: (0) entire; (1) produced into a lamina. State 1 is a synapomorphy for *Austromonticola*, but a reversal to state 0 has occurred in *A. postinventus* and *A. inflatus* (ci = 0.5, ri = 0.75).

21. Ventrite 5 of females, form of apex: (0) entire or only slightly emarginate; (1) strongly emarginate, flanked by horns. State 1 is a synapomorphy for the *A. atriarius*–*A. mataura* clade (ci = 1, ri = 1).

22. Female genitalia, presence of rectal valve (Lyal and Favreau 2015): (0) absent; (1) present as a crimped ring. State 0 is inferred on this tree to be the derived state and shared by all members of the *A. caelibatus*–*A. mataura* clade. (ci = 0.33, ri = 0.5).

23. Female genitalia, length/height ratio of distal gonocoxite: (0) long and slender, ratio greater than 1.8; (1) stout, ratio less than 1.5. State 1 is a synapomorphy for the *A. caelibatus*–*A. inflatus* clade (ci = 1, ri = 1). The unknown female of *A. caelibatus* is predicted by this phylogeny to possess state 1.

24. Female genitalia, number of sclerites in the bursa copulatrix: (0) absent; (1) one present; (2) two present. This cladogram infers that a single bursal sclerite is the plesiomorphic state, which is shared by all species of *Austromonticola*. State 0 is an apomorphy for *Brachyolus punctatus*, while state 2 is convergently present in *Inophloeus sternalis* and *Irenimus parilis* (ci = 1, ri = 1).

25. Female genitalia, form of vagina: (0) unsclerotised; (1) sclerotised. State 1 is a character uniting all species of *Austromonticola* but shared with *Brachyolus punctatus* and the undescribed genus (ci = 0.33, ri = 0.33).

26. Female genitalia, caudal shape of sclerotised rods on proximal gonocoxite: (0) straight in ventral view; (1) curved inwardly in ventral view. State 1 has arisen twice, in *A. furcatus*–*A. mataura* and apparently independently in *A. inflatus* (ci = 0.5, ri = 0.5).

27. Female genitalia, shape of sclerotised rods on proximal gonocoxite: (0) tapering proximally; (1) broadening proximally; (2) strongly broadening proximally, multiply divided. State 1 is a synapomorphy for *Austromonticola* (ci = 1, ri = 1).

28. Female genitalia, position of sclerotised rods on proximal gonocoxite in lateral view: (0) median; (1) ventral. State 1 unites all species of *Austromonticola*, but is shared with *Zenagraphus metallescens* and the undescribed genus (ci = 0.5, ri = 0.67).

29. Penis, apex in dorsal view: (0) acute; (1) sagittate; (2) truncate. State 0 is the usual form in *Austromonticola*, but state 1 has arisen twice, in *A. planulatus* and *A. rotundus* (ci = 0.5, ri = 0).

30. Penis, curvature in lateral view: (0) even from base to apex, maximum height near middle; (1) largely confined to base, maximum height near basal 1/3. The plesiomorphic state 1 is present in all species of *Austromonticola*. State 0 is a homoplasious synapomorphy for *Brachyolus punctatus* + *Irenimus parilis* and *Zenagraphus metallescens* + the undescribed genus (ci = 0.5, ri = 0.67).

31. Penis, form of ostial region: (0) tubular, unmodified; (1) thickened to form sclerotised crest (Fig. 40). State 1 is a synapomorphy for the *A. atriarius*–*A. mataura* clade (ci = 1, ri = 1).

32. Penis, length of temone in relation to length of pedon in lateral view: (0) longer than pedon; (1) shorter than pedon, but longer than 0.7 times length of pedon; (2) shorter than 0.7 times length of pedon. State 2 is a homoplasious character that unites the members of the *A. caelibatus*–*A. inflatus* clade but shared with the undescribed genus (ci = 0.5, ri = 0.5).

33. Male genitalia, shape of hemisternites 8: (0) roughly quadrate; (1) roughly triangular. The plesiomorphic state 1 is present in all species of *Austromonticola*. State 0 unites *Brachyolus punctatus* + *Irenimus parilis*, but is convergently present in *Zenagraphus metallescens* (ci = 0.5, ri = 0.5).

The species of *Austromonticola* are united by three unambiguous synapomorphies, scale structure (character 2), the obsolete striae at the elytral apex (character 19), and the proximally widening gonocoxal rods (character 27).

In *Austromonticola* there are two strongly supported clades, one consisting of the larger species *A. caelibatus*, *A. postinventus* and *A. inflatus* (the *A. inflatus* clade) and the other consisting of the smaller species possessing metanepisterna with three rows of pappolepidia, a penis with an ostial crest and ventrite 5 with a strongly emarginate apex in the females, *A. atriarius*, *A. furcatus* and *A. mataura* (the *A. mataura* clade). Grouped with the latter clade is *A. rotundus*; however, the support for this relationship is weak, and the sagittate apex of the penis shared with *A. planulatus* hints at a possible relationship. Together, *A. planulatus* and *A. rotundus* provide a transitional series between the distinctly different *A. inflatus* clade and the *A. mataura* clade.

## Discussion

### Development of ventrites in females

The highly modified ventrites in many species of *Austromonticola* are a particularly fascinating feature of the genus. There is a range of developments of the posterior margin of ventrite 4 of the females. No laminae are found in *A. inflatus* and *A. postinventus*, rather the posterior margin of ventrite 4 is recurved anteriad. A short lamina with a wide emargination is present in *A. planulatus*. Long bifurcate laminae are found in *A. mataura* and *A. atriarius*, while *A. furcatus* has a broader lamina with a deep emargination. Finally, *A. rotundus* has a long, broad lamina with a shallow emargination. The unknown female of *A. caelibatus* is predicted in the phylogenetic tree inferred above to be lacking a lamina, but it is equally parsimonious to infer a lamina being present in *A. caelibatus*, given the basal position of the species in the clade. The form of the lamina in this species would be of interest. This range of development make *Austromonticola* a suitable system for investigating the function of these laminae. Two hypotheses are presented in detail here.

### Preparation of oviposition sites

The first hypothesis is that these ventral structures assist in the preparation of oviposition sites in cushion plants. The cushion vegetational form is a distinctive feature of New Zealand alpine plants, such as *Raoulia* and *Phyllachne*. This growth habit presents densely packed foliage underlain by a peaty layer formed by decaying leaves still attached to the plant (Cockayne 1921). In this hypothesis, the long abdominal laminae and apical horns of *A. mataura* and related species are used to force aside the foliage of cushion plants to allow the ovipositor to extend into the underlying layer to deposit the eggs (Fig. 130). This model of function may explain the emargination present in the lamina, its correspondence with the horns around the genital orifice, the correlation of these structures with a long, flexible ovipositor that can be extended to 3/4 of the weevil's body length, and the damaged apex of the lamina in the specimen of *A. atriarius* (Fig. 100). Predictions made by this model are that oviposition occurs while the female is sitting on top of the cushion plant, that females thus exposed will exhibit disruptive coloration and that eggs and early-instar larvae will be found in the centre of cushions, feeding on the peaty material beneath the foliage.

This hypothesis also provides an explanation for the recurved margin of ventrite 4 of *A. inflatus* and *A. postinventus*. In these species, it is hypothesised that the form of ventrite 4 allows maximum flexion of ventrite 5, which assists in ovipositing under the side of the cushion plants where the plant meets the surrounding substrate (Fig. 131). This model explains the much stouter ovipositor possessed by *A. inflatus* and *A. postinventus*. Predictions of this model include that eggs and early-instar larvae will be found towards the edges of the cushions, feeding on roots in the soil, that adult specimens will be more frequently found beside cushions rather than on top of them, and will be coloured like the surrounding substrate.

The rather different laminae of *A. planulatus* and *A. rotundus* suggest different oviposition behaviours or host plants from those of the two scenarios postulated above.

### Mate hindrance

The second hypothesis is that these structures are mate hindrance devices. Mating pairs of entimine weevils are frequently encountered *in copula* in the field, and studies of their mating behaviour in captivity show that males will remain mounted on females for extended periods of time (D Watkin and SDJ Brown, unpub. data). The costs imposed by extended mounting include the energy expended in carrying males (Watson et al. 1998), potentially increased predation risk (Magnhagen 1991) and the losses involved in reduced foraging time (Stone 1995). Structures developed in *Austromonticola* females, such as elytral sutural tubercles, abdominal laminae and armature around the genital orifice, may enable prevention of unwanted mating attempts or assist in dislodging males if mounting becomes excessively prolonged. Evidence for this mechanism of sexual selection have been found in studies of water striders, which show similar exaggerated structures in females. Females of a number of species of *Gerris* Fabricius, 1794 (Heteroptera: Gerridae) possess elongate abdominal spines that decrease the duration of premating struggles, thereby decreasing energetic costs to the females (Arnqvist and Rowe 1995).

The two hypotheses presented above are not necessarily mutually exclusive. These two selection pressures may act synergistically, which may explain the rapid evolution of these structures. Further observations of oviposition and mating behaviour of *Austromonticola*, combined with experiments manipulating the form of the laminae, will be required to evaluate these hypotheses.

Other, alternate hypotheses for these ventral structures could include male stimulation during mating, pre-copulatory species recognition signals to prevent hybridisation, assisting the retraction of the ovipositor after oviposition has been completed and providing an area for sensory organs to determine optimum oviposition sites.

### Modified ventrites in other weevils

Although unusual, the highly modified ventrites of *Austromonticola* females are not unique. In the New Zealand context, modified ventrites are also known in species of *Chalepistes* (e.g. *C. dugdalei* (Barratt & Kuschel, 1996), *C. curvus* (Barratt & Kuschel, 1996) and *C. patricki* (Barratt & Kuschel, 1996)) and *Nicaeana*, which have medial laminae on ventrite 4 or various swellings on ventrite 5 (Barratt and Kuschel 1996). Species of *Platyacus* Faust, 1897 (Celeuthetini) in the Solomon Islands have the posterior margin of ventrite 4 developed into a medial projection or a trifurcate lamina (Tanner 1969; Marshall 1956). Most species of the endemic Mauritian genus *Syzygops* Schönherr, 1826 (Ottistirini) have simple ventrites in both sexes (Williams 2000), but several have modifications that include a large, trifurcate lamina on ventrite 4 (*S. insignis* Williams, 2000), a thin median projection on ventrite 4 (*S. ornatus* Williams, 2000), and ventrite 4 being almost completely invaginated (*S. vinsoni* Hustache, 1939). Some populations of *Trichalophus caudiculatus* (Fairmaire, 1886) (Tropiphorini) found in the Chinese Himalayas possess a bifurcate lamina on ventrite 4 (Grebennikov 2015), which has a similar shape to that of *A. furcatus* (Fig. 102). Species of *Leptomias* Faust, 1886 (Tanymecini) from montane Kashmir possess variable numbers and shapes of abdominal laminae; whereas *L. costatus* (Faust, 1897) has a lamina on ventrite 4 only, *L. montanus* (Aslam, 1969), *L. fletcheri* (Aslam, 1969) and *L. rufus* (Aslam, 1969) have a lamina on ventrite 3 in addition to one on ventrite 4 (Aslam 1969). The species possessing these laminae were represented only by females, and there is no indication that Aslam (1969) recognised these structures as sexually dimorphic. Laminae are also known from some Himalayan species of *Leptomias* (Li Ren pers. comm.) and Central American *Sciomias* Sharp, 1911 (Sciaphilini) (R. Anderson pers. comm.), but no details have been published. Abdominal laminae are also known in the subfamily Cyclominae, with females of the *Nestrius bifurcus* Kuschel, 1964 group of species having laminae formed at the posterior margin of ventrite 3 (Kuschel 1964).

The cushion growth form is a feature of alpine vegetation worldwide, and is prevalent in the Himalayas (Chen et al. 2015, Dolezal et al. 2016), where several of the weevil species discussed above are found. Additionally, *Syzygops* is associated with ferns, which frequently present a dense rhizome mat. These observations lend support to the first hypothesis detailed above, which posits that abdominal laminae assist with preparation of oviposition sites in close-packed vegetational structures. Further investigation of the oviposition behaviour of these weevils will be necessary to accurately evaluate this hypothesis. It also predicts that weevils displaying abdominal laminae will be found in other regions where cushion vegetation is present, such as Tasmania (Gibson and Kirkpatrick 1985), the Andes (Molina-Montenegro et al. 2006) and Siberia (Volkov and Volkova 2015).

### Relationships

The morphological phylogeny is largely consistent with the molecular data, in that both indicate a close relationship between *A. furcatus*, *A. atriarius* and *A. mataura*. However, the position of *A. rotundus* in the morphology-based tree, placed as the sister taxon of the *A. mataura* clade, is not supported by the molecular data. The overall signal from the molecular data is that *A. rotundus* is the most distant of all the species for which DNA sequences were obtained, however no consensus was gained regarding its nearest relative. Results from COI and 28S are surprising. In the analysis of COI, *A. inflatus* was nearest to *A. rotundus*, whereas *A. inflatus* has the same 28S sequence as *A. atriarius* and *A. furcatus*. These results serve to bolster confidence that *A. inflatus*, *A. postinventus* and *A. caelibatus* are congeneric with the other members of the genus. Obtaining DNA sequences from the other species in the genus, especially the morphologically distinct *A. planulatus*, will be important for further insights into the relationships of species in the genus.

### New Zealand alpine weevil fauna

*Austromonticola* is one of a number of weevils that inhabit the montane regions of New Zealand. Other weevil genera with representatives found above the treeline include *Baeosomus* Broun, 1904 (Brachycerinae), *Anagotus* Sharp, 1882, *Gromilus* Blanchard, 1853, *Liparogetus* Broun, 1915, *Lyperopais* Broun, 1893 (Cyclominae), *Lyperobius* Pascoe, 1876, “*Crisius*” Pascoe, 1876 (Molytinae), *Eugnomus* Schönherr, 1847, *Oreocalus* May 1993, *Pactolotypus* Broun, 1909, *Stephanorhynchus* White, 1846 (Curculioninae: Eugnomini), *Peristoreus* and *Simachus* (Curculioninae: Storeini). However, the Entiminae are best represented, with 13 genera (*Austromonticola*, *Sargon* Broun, 1903, *Inophloeus*, *Chalepistes*, *Catoptes*, *Nicaeana*, *Haplolobus*, *Zenagraphus*, *Neoevas* Broun, 1921, and four undescribed genera) having species found primarily or solely in montane environments over 1000 m in elevation.

This diverse community is at apparent odds with the young geological age of the environment. The Southern Alps began rising around 5 million years ago (Sutherland 1996). The Old Man Range is older, with uplift estimated to have begun in the middle Miocene (c. 15 mya) (Craw et al. 2012). The Hawkdun and Kirkliston Ranges are estimated to have begun rising in the late Miocene (Youngson et al. 1998; Forsyth 2001). Prior to this time, the landscape of the Central Otago region is inferred to have been a low-relief basin, dominated by the large, freshwater Lake Manuherikia (Mildenhall 1989).

A geobiological model of the origin of the New Zealand alpine flora posited by Heenan and McGlone (2013) infers that a sizable component of the modern flora is derived from the community of plants that inhabited infertile and boggy lowland environments. It is noteworthy that one of the plant genera mentioned explicitly by Heenan and McGlone (2013) as providing evidence for their model is *Phyllachne*, upon which several species of alpine Entiminae have been collected (SDJ Brown pers. obs.). This model predicts that closely related species or genera may be found in lowland bogs. Unfortunately, these habitats have been greatly diminished as a result of agricultural intensification. However, there remain relatively intact remnant wetland systems in Southland that have a similar vegetation community to alpine bogs (McGlone 2009) and which may harbour sister taxa of alpine specialists. An example of this scenario is the crambid moth *Orocrambus thymiastes* Meyrick, 1901, which is found in alpine boggy regions, but has a population in the Awarua-Waituna Wetlands (Gaskin 1975).

An alternative possibility for the origin of the New Zealand alpine biota is dispersal from alpine regions in Australia, South America or the Northern Hemisphere. These areas have been the main sources for the majority of New Zealand alpine plant radiations (Winkworth et al. 2005). Dispersal from alpine areas elsewhere is an unlikely scenario for New Zealand's alpine weevils, as most of the genera are New Zealand endemics, with no close relatives elsewhere. However, little work has been done on the relationships of New Zealand weevils to other world faunas, and until these studies have been done, the dispersal hypothesis will remain untested.

Research into the mechanisms by which these weevil lineages have adapted to alpine environments, as has been investigated in other New Zealand alpine insects (Wharton 2011; Dunning et al. 2014), will be useful to inform further hypotheses of the origin of the New Zealand alpine weevil fauna.

### Conservation significance

The localised distribution of most of the species of *Austromonticola* place them within the Naturally Uncommon (Range Restricted) threat classification category (Townsend et al. 2008). All species already have significant portions of their range administered by DOC as Stewardship Areas. The main threats to these species are likely to be predation by introduced mammals (O'Donnell et al. 2017) and encroachment of weeds. Non-target parasitism by adventive wasps is also a potential threat (Barlow et al. 2004; Barratt et al. 2007). All of these threats are expected to increase due to climate change (Halloy and Mark 2003). The much larger distribution of *A. rotundus* results in it being given the classification of Not Threatened.

## Conclusion

Additional research into the biology, behaviour and physiology of the species of *Austromonticola* described here will offer insight into the function of the exaggerated abdominal structures of the females, and into processes by which sexual selection accelerates speciation. Further exploration and collecting, especially in areas such as the Mt Teviot/Manorburn region in Central Otago, the Pisa Range, Dunstan Mountains and Mt Aspiring National Park, will be vital for discovering additional species in the genus, which will provide further data for evaluating hypotheses of the role of historical contingency and environmental pressures on the evolution of alpine insects.

## Plates

**Figures 1–8.**
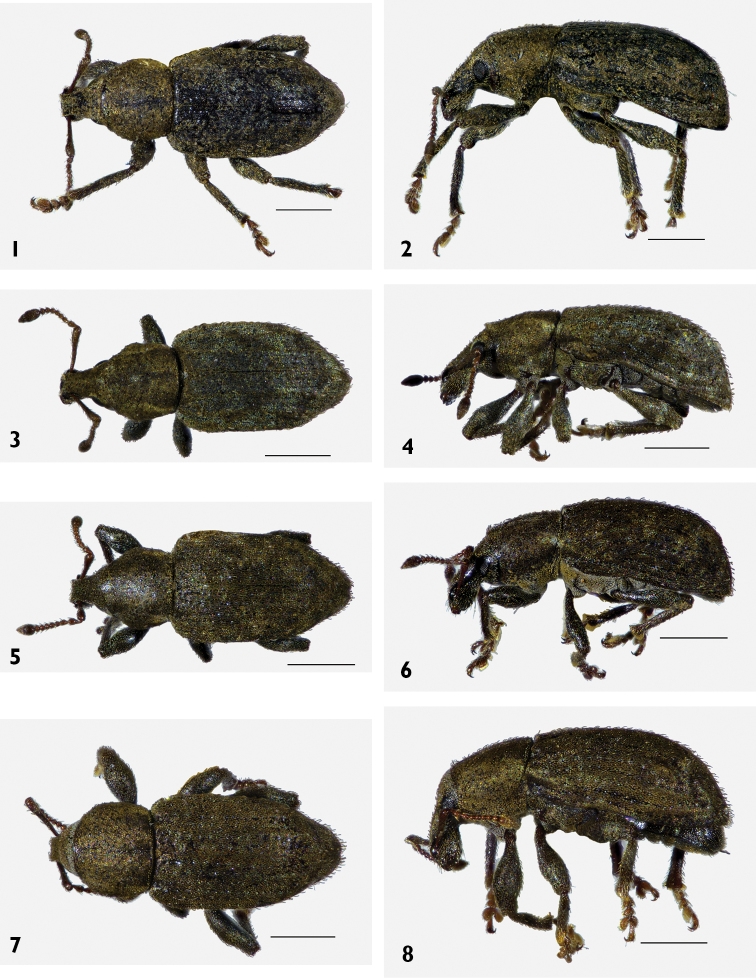
Habitus photographs of *Austromonticola* males. **1, 2** *A. atriarius* **3, 4** *A. mataura* **5, 6** *A. furcatus* **7, 8** *A. rotundus*. Scale bars = 1 mm.

**Figures 9–16.**
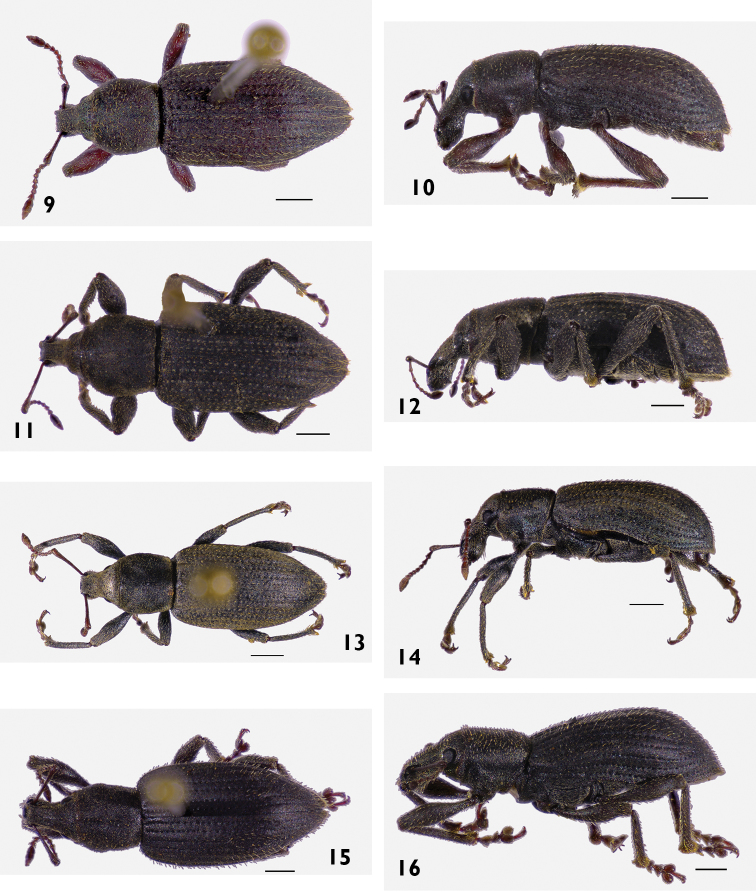
Habitus photographs of *Austromonticola* males. **9, 10** *A. postinventus* **11, 12** *A. planulatus* **13, 14** *A. inflatus* **15, 16** *A. caelibatus*, holotype. Scale bars = 1 mm.

**Figures 17–24.**
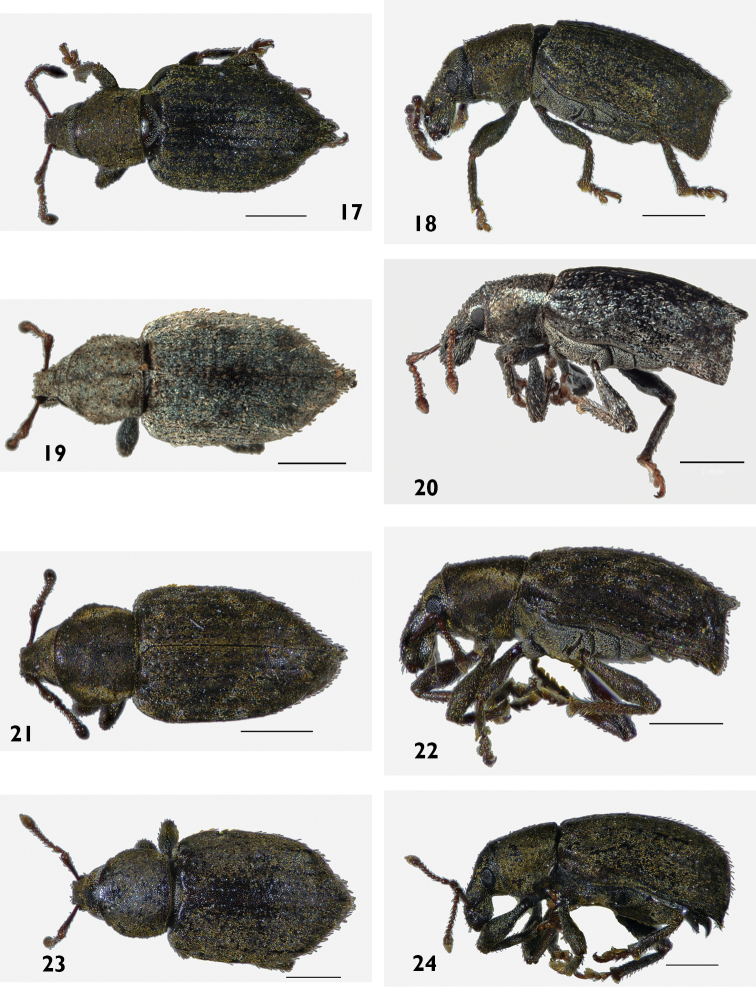
Habitus photographs of *Austromonticola* females. **17, 18** *A. atriarius* **19, 20** *A. mataura* **21, 22** *A. furcatus* **23, 24** *A. rotundus*. Scale bars = 1 mm.

**Figures 25–30.**
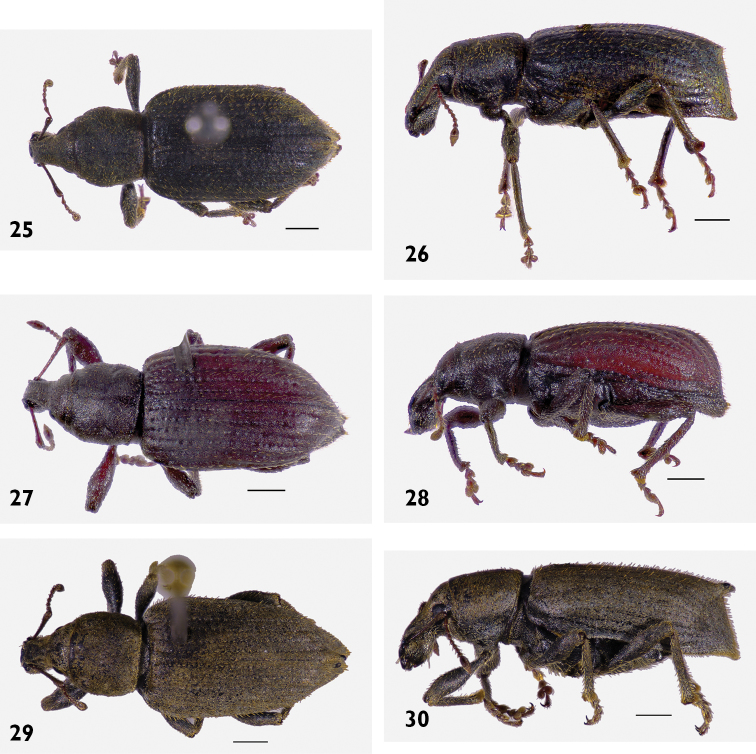
Habitus photographs of *Austromonticola* females. **25, 26** *A. postinventus*, holotype **27, 28** *A. planulatus*, holotype **29, 30** *A. inflatus*, holotype. Scale bars = 1 mm.

**Figures 31–32.**
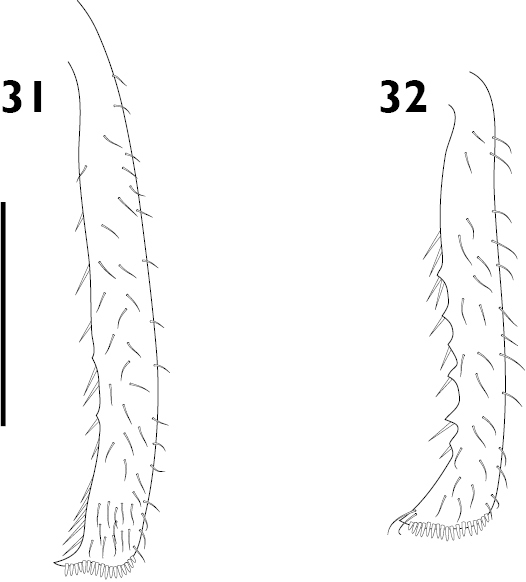
Left protibia, anterior view. **31** *Austromonticola inflatus*, holotype **32** *Austromonticola planulatus*, holotype. Scale bar = 1 mm.

**Figures 33–34.**
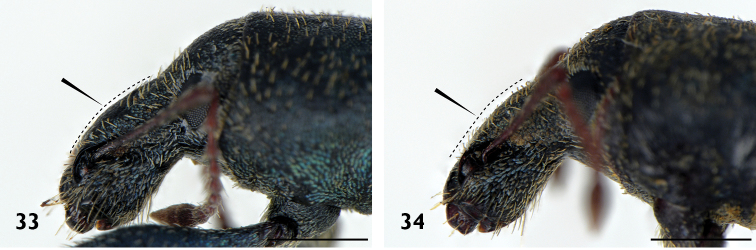
Rostrum, lateral view. **33** *Austromonticola inflatus*, arrow and dashed line indicates convex epifrons **34** *Austromonticola postinventus*, holotype, arrow and dashed line indicates flattened. Scale bars = 1 mm.

**Figures 35–36.**
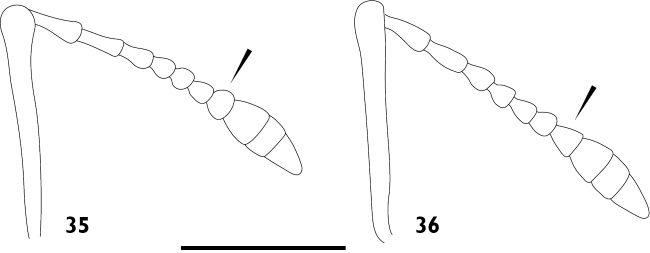
Left antenna, anterior view. **35** *Austromonticola inflatus*, holotype **36** *Austromonticola postinventus*, holotype. Arrows indicate funicle segment 7. Scale bar = 1 mm.

**Figures 37–38.**
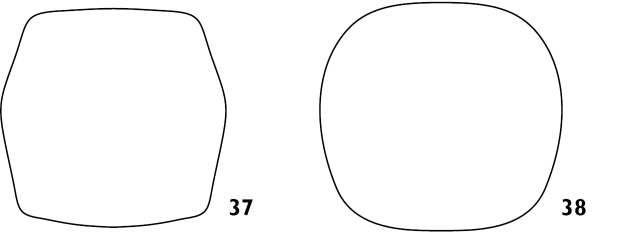
Pronotum, dorsal view. **37** *Austromonticola furcatus* **38** *Austromonticola rotundus*.

**Figures 39–45.**
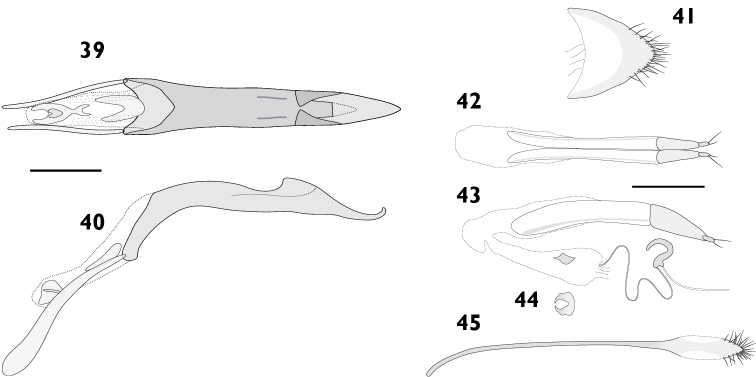
Genitalia of *Austromonticola atriarius*. **39** penis, dorsal view **40** aedeagus, lateral view **41** female tergite 8, dorsal view **42** ovipositor, dorsal view **43** ovipositor and spermatheca, lateral view **44** bursal sclerite, ventral view **45** female sternite 8, ventral view. Scale bars = 0.5 mm; **39–40** at same scale; **41–45** at same scale.

**Figures 46–49.**
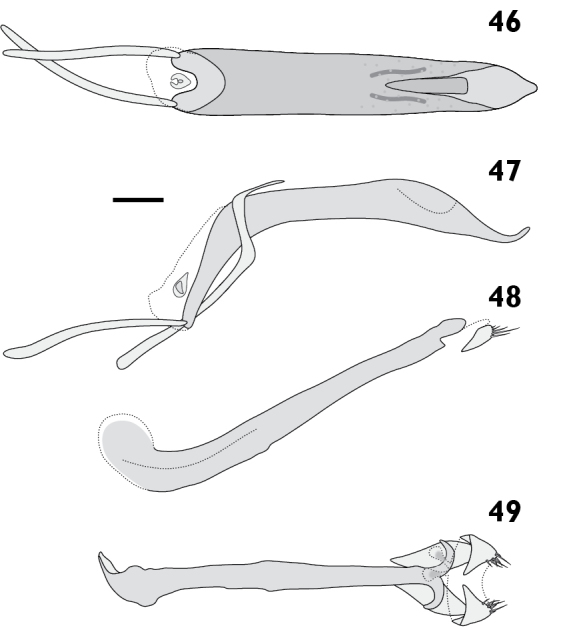
Genitalia of *Austromonticola caelibatus*. **46** penis, dorsal view **47** aedeagus, lateral view **48** male hemisternites 8 and spiculum gastrale, lateral view (membrane between hemisternites 8 and basal plate indicated) **49** male hemisternites 8 and spiculum gastrale with basal plate, ventral view. Scale bar = 0.5 mm.

**Figures 50–57.**
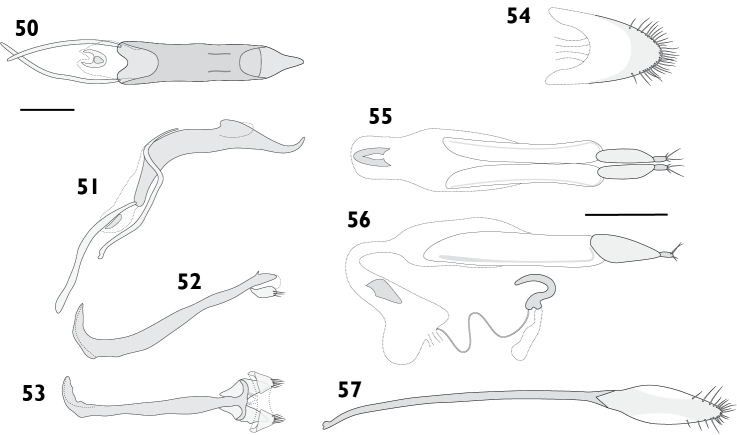
Genitalia of *Austromonticola furcatus*. **50** penis, dorsal view **51** aedeagus, lateral view **52** male hemisternites 8 and spiculum gastrale, lateral view (membrane between hemisternites 8 and basal plate indicated) **53** male hemisternites 8 and spiculum gastrale with basal plate, ventral view **54** female tergite 8, dorsal view **55** ovipositor, dorsal view **56** ovipositor and spermatheca, lateral view **57** female sternite 8, ventral view. Scale bars = 0.5 mm; **50–53** at same scale; **54–57** at same scale.

**Figures 58–66.**
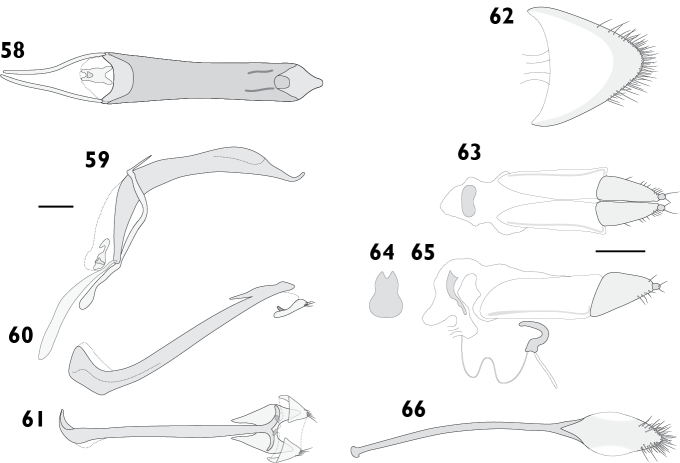
Genitalia of *Austromonticola inflatus*. **58** penis, dorsal view **59** aedeagus, lateral view **60** male hemisternites 8 and spiculum gastrale, lateral view (muscles between hemisternites 8 and basal plate indicated) **61** male hemisternites 8 and spiculum gastrale with basal plate, ventral view **62** female tergite 8, dorsal view **63** ovipositor, dorsal view **64** bursal sclerite, anterior view **65** ovipositor and spermatheca, lateral view **66** sternite 8, ventral view. Scale bars = 0.5 mm; **58–61** at same scale; **62–66** at same scale.

**Figures 67–75.**
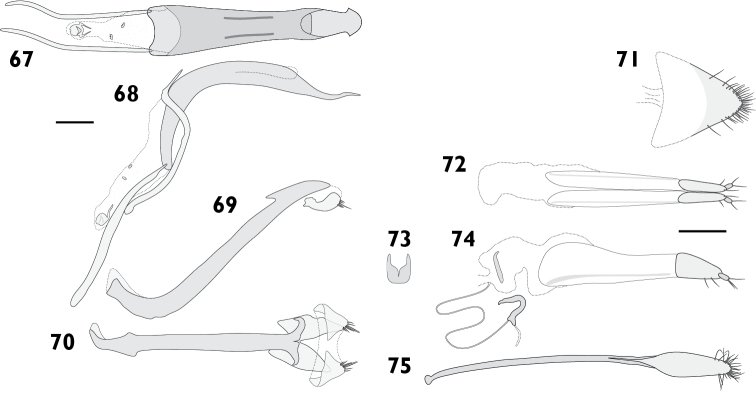
Genitalia of *Austromonticola planulatus*. **67** penis, dorsal view **68** aedeagus, lateral view **69** male hemisternites 8 and spiculum gastrale, lateral view (muscles between hemisternites 8 and basal plate indicated) **70** male hemisternites 8 and spiculum gastrale with basal plate, ventral view **71** tergite 8, dorsal view **72** ovipositor, dorsal view **73** bursal sclerite, anterior view **74** ovipositor and spermatheca, lateral view **75** female sternite 8, ventral view. Scale bars = 0.5 mm; 67–70 at same scale; 71–75 at same scale.

**Figures 76–84.**
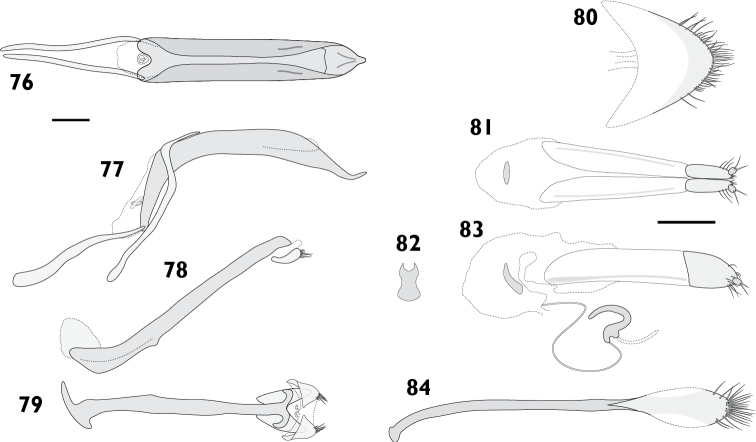
Genitalia of *Austromonticola postinventus*. **76** penis, dorsal view **77** aedeagus, lateral view **78** male hemisternites 8 and spiculum gastrale, lateral view (muscles between hemisternites 8 and basal plate indicated) **79** male hemisternites 8 and spiculum gastrale with basal plate, ventral view **80** female tergite 8, dorsal view **81** ovipositor, dorsal view **82** bursal sclerite, anterior view **83** ovipositor and spermatheca, lateral view **84** female sternite 8, ventral view. Scale bars = 0.5 mm; **75–79** at same scale; **80–84** at same scale.

**Figures 85–92.**
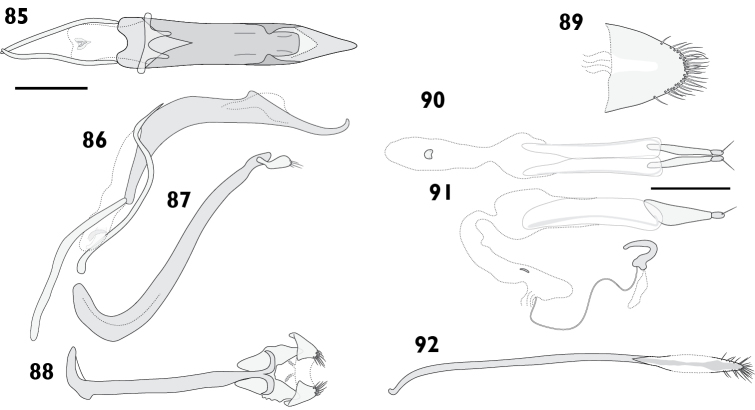
Genitalia of *Austromonticola mataura*. **85** aedeagus, dorsal view **86** aedeagus, lateral view **87** male hemisternites 8 and spiculum gastrale, lateral view (muscles between hemisternites 8 and basal plate indicated) **88** male hemisternites 8 and spiculum gastrale with basal plate, ventral view **89** female tergite 8, dorsal view **90** ovipositor, dorsal view **91** ovipositor and spermatheca, lateral view **92** female sternite 8, ventral view. Scale bars = 0.5 mm; **85–88** at same scale; **89–92** at same scale.

**Figures 93–99.**
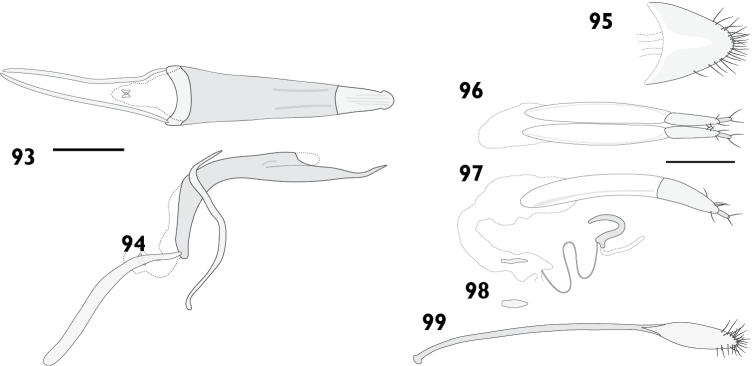
Genitalia of *Austromonticola rotundus*. **93** penis, dorsal view **94** aedeagus, lateral view **95** female tergite 8, dorsal view **96** ovipositor, dorsal view **97** ovipositor and spermatheca, lateral view **98** bursal sclerite, ventral view **99** female sternite 8, ventral view. Scale bars = 0.5 mm; **93–94** at same scale; **95–99** at same scale.

**Figures 100–105.**
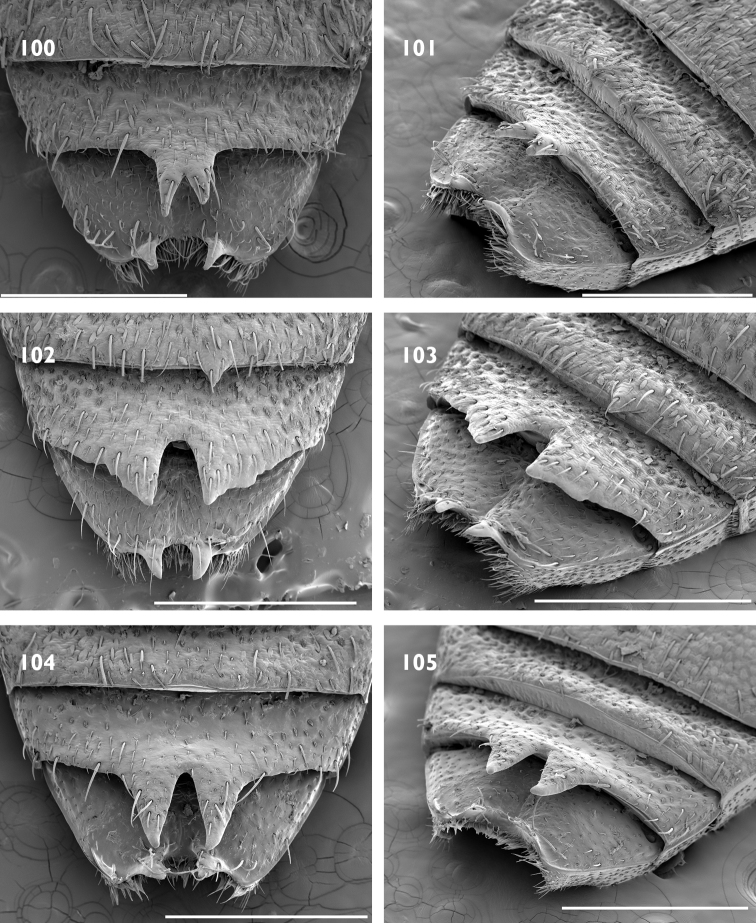
SEM photographs of abdominal ventrites 4 and 5 of *Austromonticola* females. **100, 101** *A. atriarius* **102, 103** *A. furcatus* **104, 105** *A. mataura*. Left: ventral view. Right: ventroposterolateral view. Scale bars = 0.5 mm.

**Figures 106–111.**
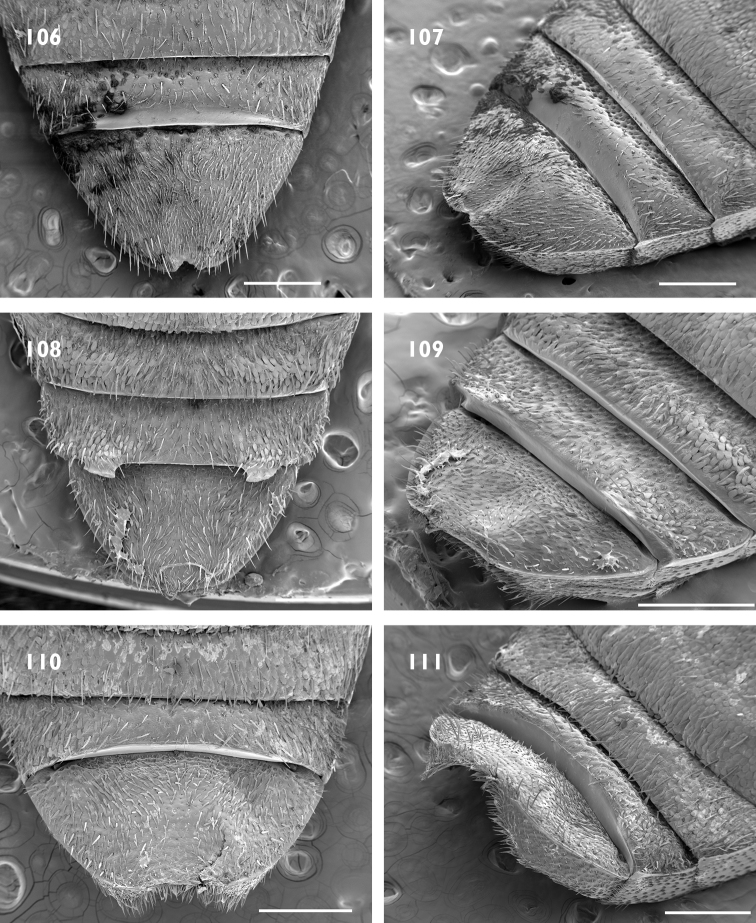
SEM photographs of abdominal ventrites 4 and 5 of *Austromonticola* females. **106, 107** *A. inflatus*, holotype **108, 109** *A. planulatus*, holotype **110, 111** *A. postinventus*, holotype. Left: ventral view. Right: ventroposterolateral view. Scale bars = 0.5 mm.

**Figures 112–113.**
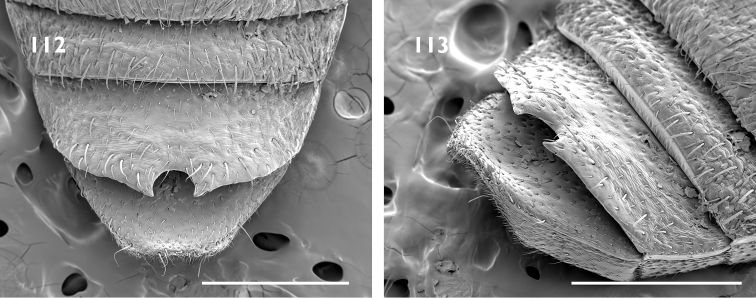
SEM photographs of abdominal ventrites 4 and 5 of female *Austromonticola rotundus*. **112** ventral view **113** ventroposterolateral view. Scale bars = 0.5 mm.

**Figure 114.**
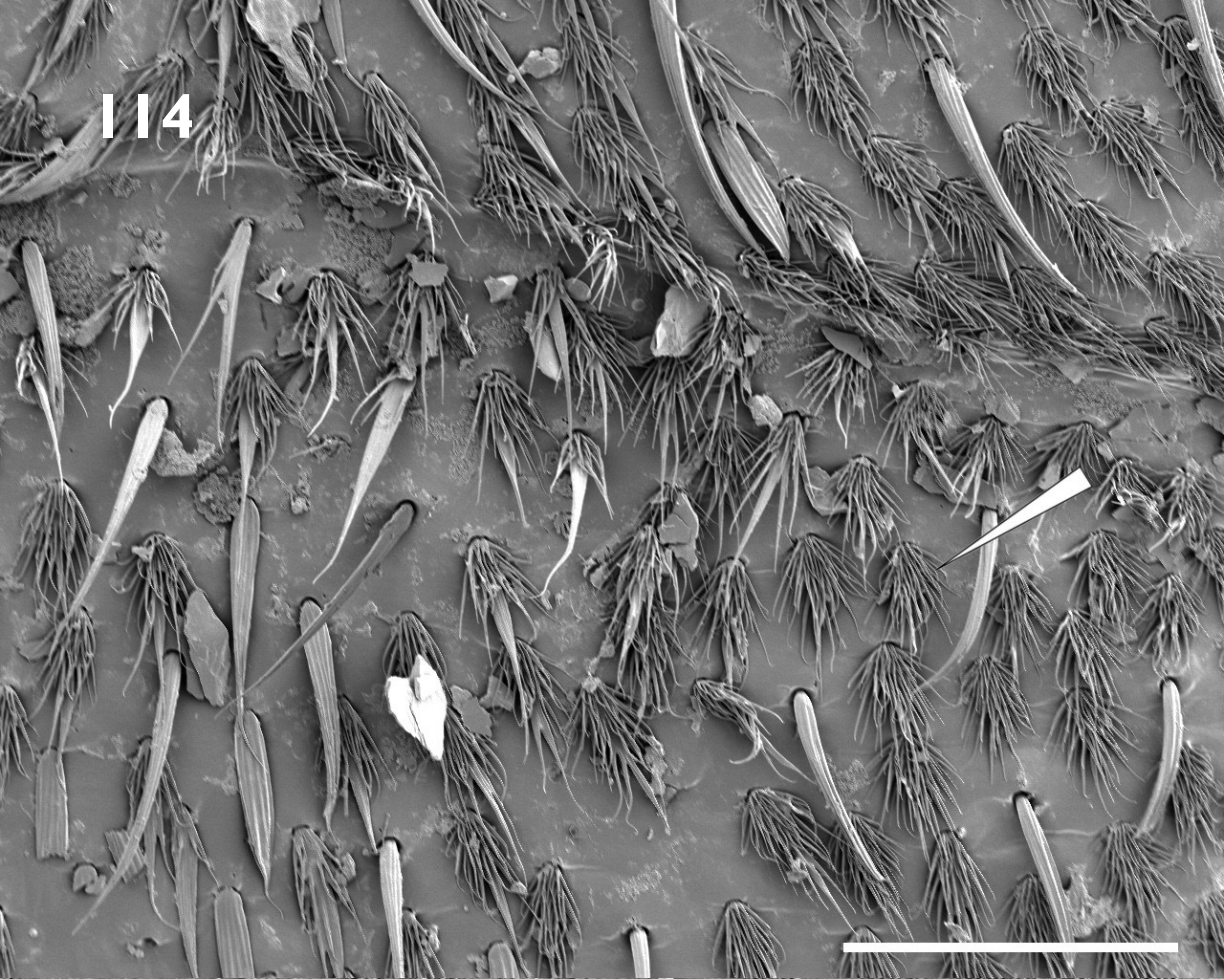
SEM photograph of *Austromonticola furcatus* abdominal ventrite 2 showing pappolepidia (arrowed). Scale bar = 0.1 mm.

**Figure 115.**
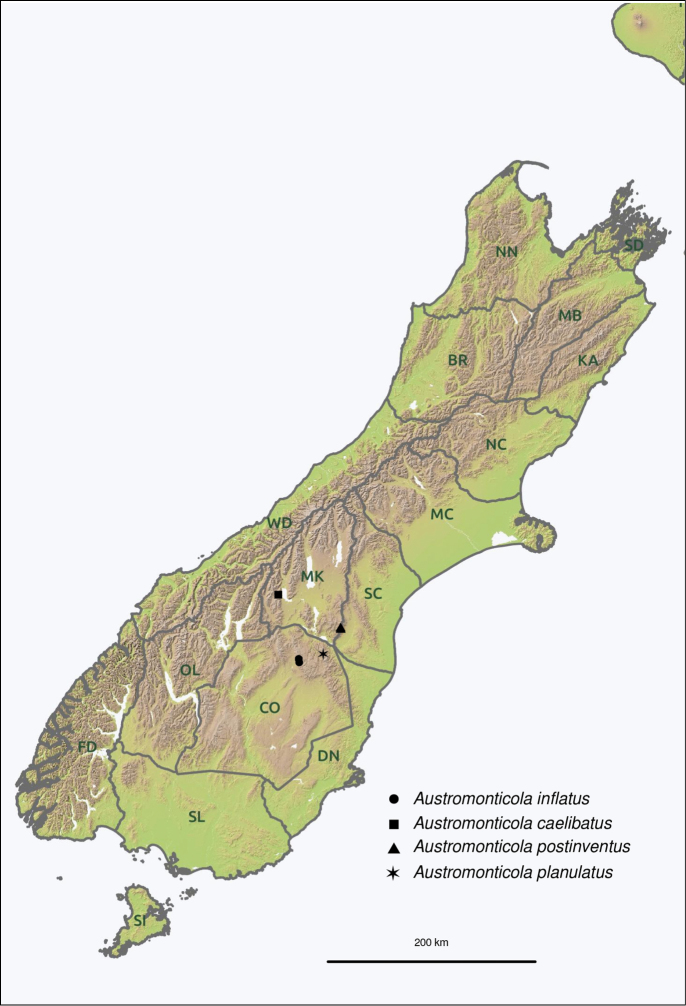
Distributions of *Austromonticola inflatus* (circles), *A. caelibatus* (squares), *A. postinventus* (triangles) and *A. planulatus* (stars).

**Figure 116.**
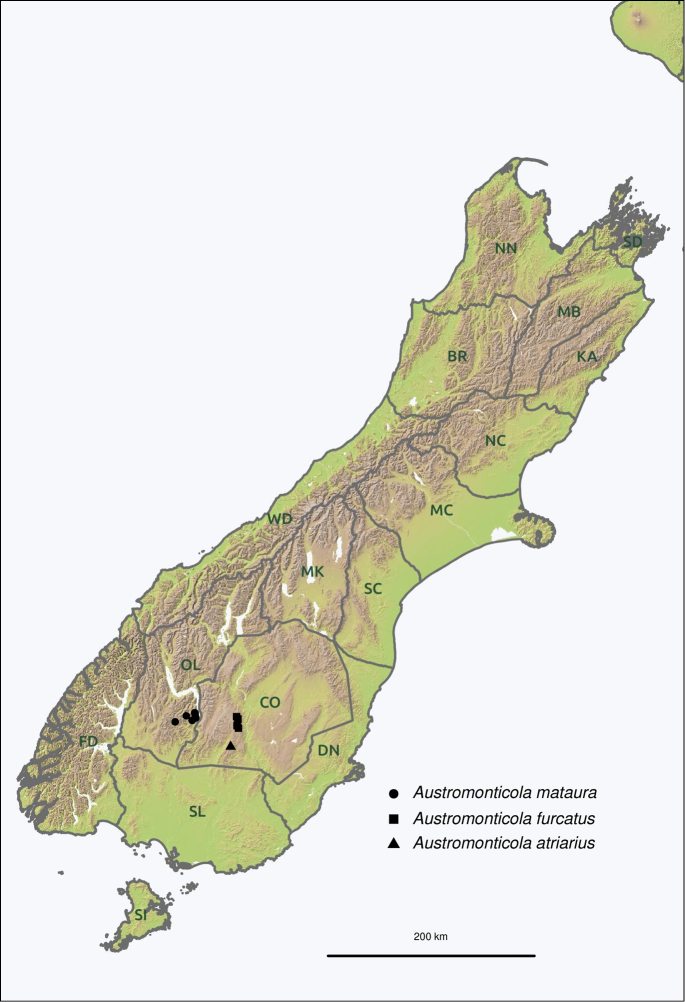
Distributions of *Austromonticola mataura* (circles), *A. furcatus* (squares) and *A. atriarius* (triangles).

**Figure 117.**
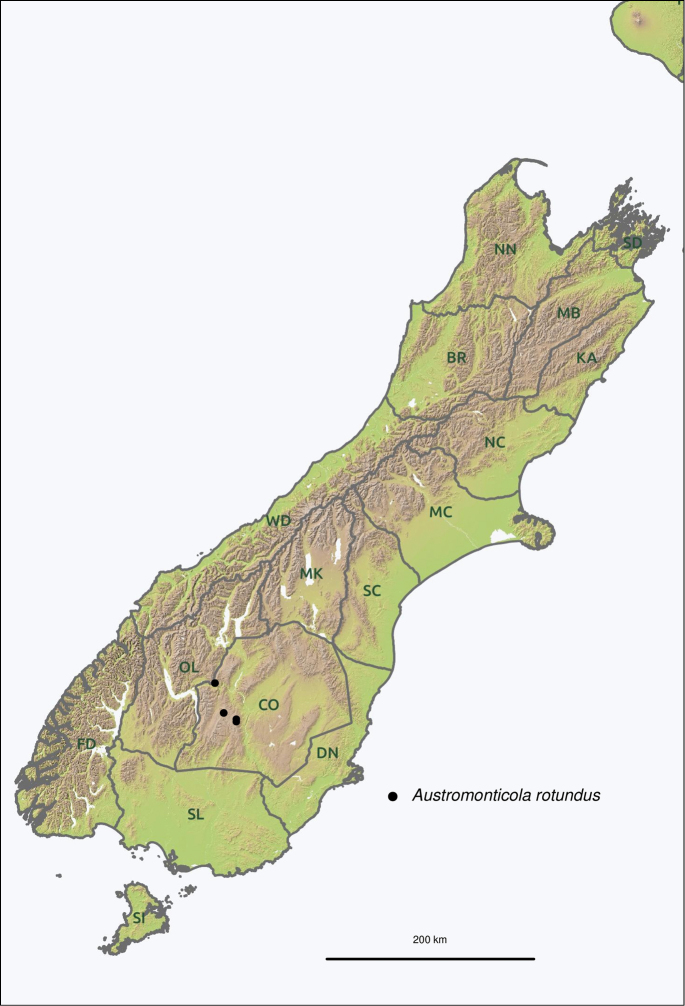
Distribution of *Austromonticola rotundus*.

**Figures 118–119.**
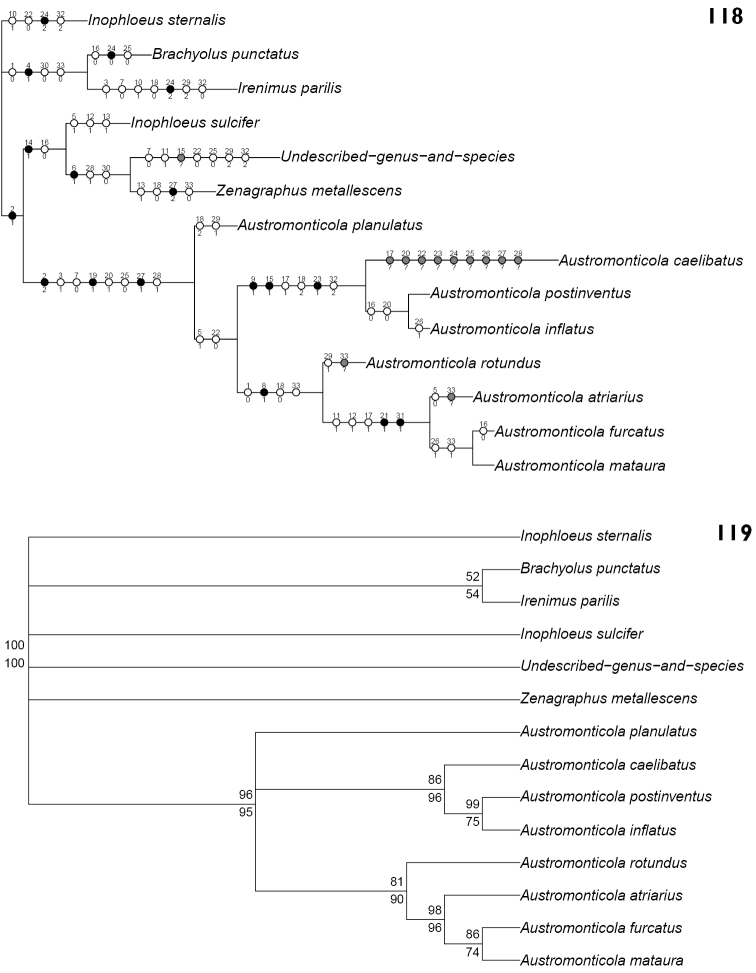
Cladograms showing phylogenetic relationships between *Austromonticola* species as inferred from morphological data. **118** single most parsimonious tree inferred from 33 characters scored for 14 species **119** phylogenetic tree of *Austromonticola* with bootstrap values above nodes and jackknife values below. Nodes with lower than 50% support were collapsed.

**Figures 120–124.**
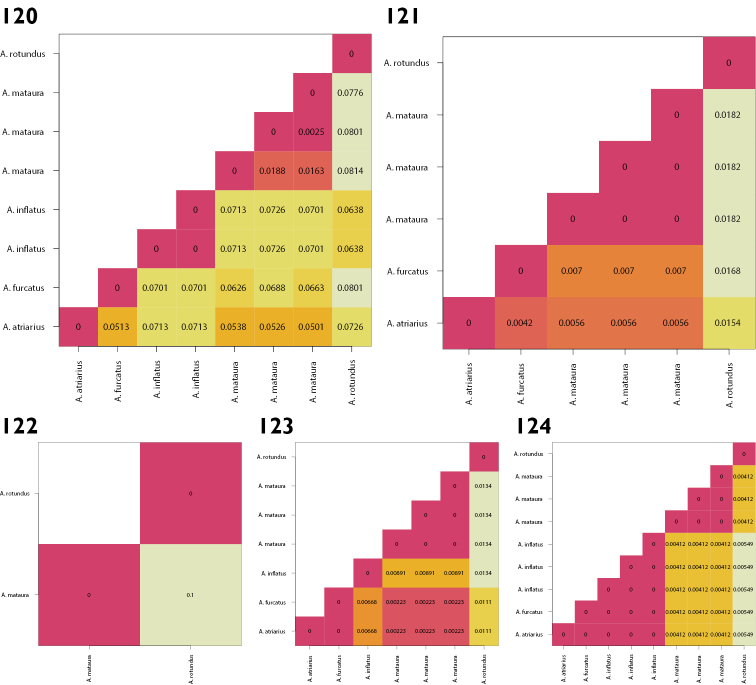
Heatmaps of the uncorrected pairwise genetic distances between *Austromonticola* specimens sampled. Lighter colours indicate greater distances. **120** the 3^'^ region of the COI mitochondrial protein-coding gene **121** CAD nuclear protein-coding gene **122** the 5^'^ (“barcoding”) region of COI **123** ArgK nuclear protein-coding gene **124** 28S nuclear ribosomal RNA gene.

**Figures 125–129.**
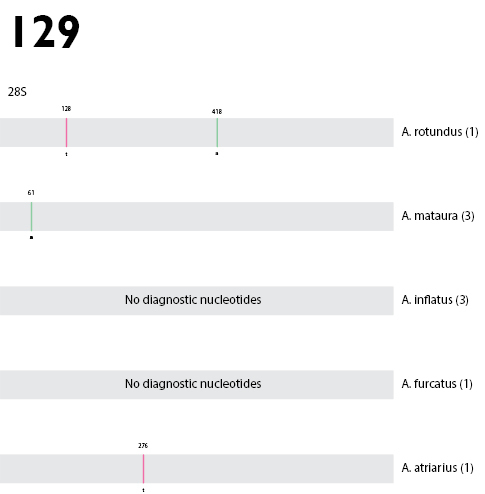
Diagnostic nucleotides within the *Austromonticola* specimens sampled. Numbers above the bars indicate the position of the diagnostic base within the alignment. Numbers in parentheses beside species names indicate the numbers of specimens included in the alignment. Letters below the bars and the colour of the vertical bar indicate the value of the diagnostic nucleotide. **125** the 5^'^ (“barcoding”) region of the COI mitochondrial protein-coding gene **126** the 3^'^ region of COI **127** CAD nuclear protein-coding gene **128** ArgK nuclear protein-coding gene **129** 28S nuclear ribosomal RNA gene.

**Figures 130–131.**
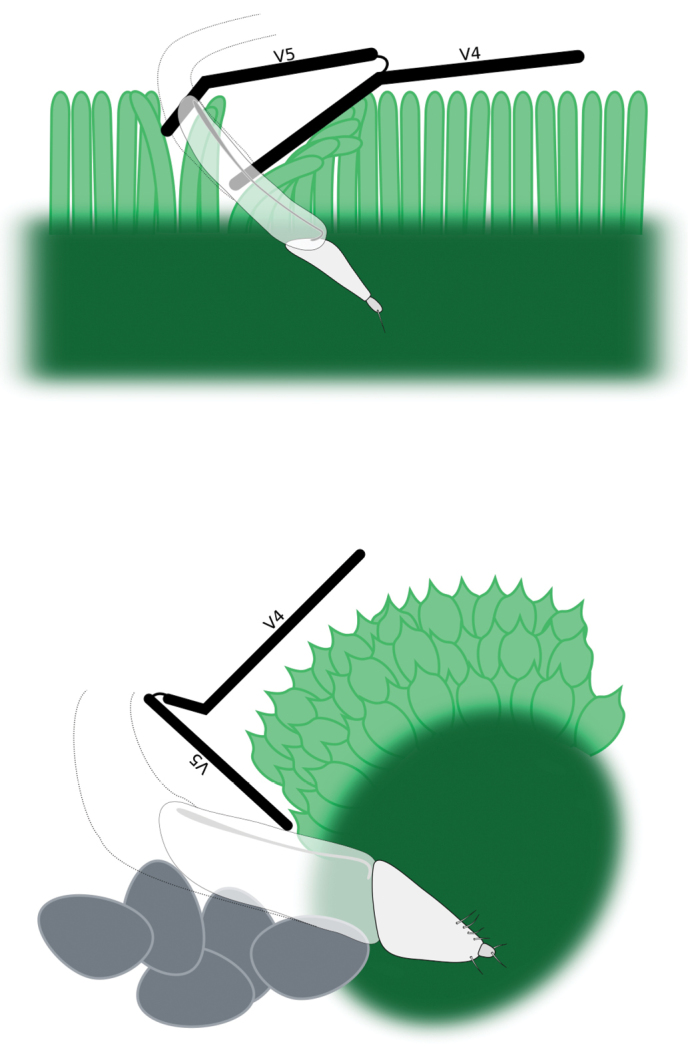
Schematic diagrams of hypothesised oviposition posture. **130** hypothesised function of lamina on ventrite 4 and horns surrounding genital orifice on ventrite 5, which force apart dense foliage of cushion plants to allow oviposition in peaty layer underneath **131** hypothesised function of recurved margin of ventrite 4 which allows maximum flexion of ventrite 5, enabling oviposition under the side of cushions between the plant and the surrounding substrate. Abbreviations: V4–ventrite 4; V5–ventrite 5. Figures not drawn to scale.

## Supplementary Material

XML Treatment for Austromonticola

XML Treatment for Austromonticola atriarius

XML Treatment for Austromonticola caelibatus

XML Treatment for Austromonticola furcatus

XML Treatment for Austromonticola inflatus

XML Treatment for Austromonticola planulatus

XML Treatment for Austromonticola postinventus

XML Treatment for Austromonticola mataura

XML Treatment for Austromonticola rotundus
